# Identification of divergent PTPN11 mutations in canine histiocytic sarcomas reveals evidence of an independent clonal origin

**DOI:** 10.1371/journal.pone.0345429

**Published:** 2026-07-20

**Authors:** Stéphanie Mottier, Armel Houel, Solveig Bouchard, Charline Bianchi, Louis Le Nezet, Richard Guyon, Stéphane Dreano, Thomas Derrien, Jerôme Abadie, Catherine André, Benoît Hédan

**Affiliations:** 1 Faculty of Medicine, University of Rennes, CNRS, IGDR, Institute of Genetics and Development of Rennes, SFR Biosit, Rennes, France; 2 Oniris, LabOniris, Nantes, France; Colorado State University, UNITED STATES OF AMERICA

## Abstract

**Background:**

Histiocytic sarcoma (HS) is a rare but highly aggressive neoplasm in humans, with limited knowledge on indicators markers and no standardized treatment. Dogs naturally develop HS, offering a powerful comparative model. Activating mutations in *PTPN11*, affecting two recurrent hotspots (E76 and G503), are the most frequent drivers of canine HS and are strongly associated with the disseminated form (DHS). While DHS is typically considered metastatic, we hypothesize that multiple independent tumor clones may arise within the same individual.

**Methods:**

We investigated clonal heterogeneity in 38 dogs affected with DHS presenting tumors in both abdominal and thoracic organs. *PTPN11* hotspot mutations were analyzed by high-sensitivity droplet digital PCR (ddPCR) to detect low-abundance variants. The frequency of divergent mutations between tumor sites was used to infer the proportion of cases arising from independent clonal origins.

**Results:**

Divergent *PTPN11* mutations were directly observed in 3 of 28 dogs with *PTPN11*-mutated tumor pairs. After correcting for the limited detectability inherent to hotspot-based analysis, we estimated that, in this cohort, up to 24–45% of DHS cases may harbor clonally independent *PTPN11* mutations in distinct tumors. In addition, analysis of somatic mutations and copy number variants by Next Generation Sequencing (NGS) in two cell lines derived from the same patient confirmed the independent origin of the clones.

**Conclusions:**

Our findings suggest that a significant numbers of dogs with DHS develop several genetically distinct HS lesions either simultaneously or within a short time interval. This need to be confirmed in larger cohorts but this clonal diversity challenges the assumption that DHS is predominantly metastatic and has major implications for prognosis, therapeutic strategies, and the use of canine HS as a comparative model for human disease. Further exploration of clonal evolution in HS is warranted to improve treatment approaches in both species.

## 1 Introduction

In humans, histiocytic sarcomas (HS) are rare yet aggressive tumors involving mature histiocytes, such as dendritic cells or macrophages [1–3]. Given their rarity, there is currently no consensus on prognostic factors or standardized treatments. Dogs are also naturally affected with HS, with a high incidence observed in certain predisposed breeds, representing an excellent spontaneous model for deciphering the genetic mechanisms involved in this devastating cancer [4]. We and others have already demonstrated that MAPK (Mitogen Activated Protein Kinase) pathway plays a crucial role in the development of canine HS similar to human HS [4–7]. This pathway is notably altered in up to 64% of the canine cases, primarily due to gain-of-function mutations of the *PTPN11* gene in nearly 50% of HS in dogs [4,5,8]. These mutations are remarkably found in the same two main hotspots 1 and 2 as in human HS, i.e., E76 and G503. Additionally, we have demonstrated that these mutations are associated with the disseminated visceral form of HS in both dogs and human [4]. In dogs, disseminated histiocytic sarcoma (DSH), by contrast with localized histiocytic sarcoma (LSH), is characterized by the presence of multiple masses occurring simultaneously at various sites of different tissues and organs [9–11]. This devastating clinical form is observed more frequently in the predisposed Bernese mountain dog breed (BMD) rather than in Flat coated Retrievers (FCR), another commonly affected canine breed [9,10,12]. The prevailing hypothesis is that DSH represents a late stage of HS after metastatic spread to organs such as spleen, lungs, lymph nodes, liver or kidneys [9]. Our previous comparative genomic hybridization (CGH) analysis supports this hypothesis by demonstrating the metastatic origin of disseminated HS in 4 out of 5 BMD cases. In the remaining case, however, distinct *PTPN11* mutations were identified in abdominal versus thoracic tumors, suggesting the possible occurrence of subclonal mutations of *PTPN11* in the late stages of the tumor progression [4]. Gaining access to numerous cases of disseminated HS in popular and frequently affected canine predisposed breeds represents a unique opportunity to explore the natural history, clonal evolution, and timing of somatic events underlying HS development. The heterogeneity observed among cancer cells within the same tumor or across different tumor sites can serve as a valuable tool to elucidate the temporal occurrence of these events.

In the present study, we investigated the heterogeneity of *PTPN11* mutations in canine DHS. Since Erich and colleagues reported that in most canine HS cases at the time of diagnosis, a single body compartment—such as the abdomen or thorax—is predominantly involved [10], we opted to examine a cohort of cases in which tumors were concurrently present in both body compartments, i.e., abdominal and thoracic organs. By droplet digital PCR (ddPCR)—a highly sensitive and precise technique that enables the detection of low-abundance mutations—, we assessed the *PTPN11* mutation status in paired lesions and quantified the frequency of clonal diversity at diagnosis in a cohort of 38 dogs with DHS involving both abdominal and thoracic sites. Additionally, whole-genome sequencing (WGS) analysis of somatic mutations and copy number variants in two cell lines derived from the same patient confirmed that this clonal diversity could be associated with the independent origin of clones.

## 2 Materials and methods

### 2.1 Sample collection and cell lines

Blood and tissue biopsy samples from dogs were collected by veterinarians, in the course of the dogs’ medical care, with the owners’ consents, through the Cani-DNA Biobank (https://igdr.univ-rennes.fr/crb-cani-dna). This study involving dog samples was approved by the Ethics Committee (CREEA - #CE07-2020-11-CA).

For 38 dogs, between 2–5 ml of blood was taken during a routine examination or during the clinical follow-up of the dog and was collected in ethylenediamine tetraacetic acid (EDTA).

Tissue biopsy samples were collected by the attending veterinarians as part of routine veterinary care, and no animals were euthanized for the purposes of this study. Thus, anesthesia and/or analgesia methods, as well as measures to alleviate suffering, were administered by the attending veterinarian as part of the proper practice of veterinary medicine. Each biopsy was divided into two parts, one was placed in RNALater solution for genomic experiments and the other was fixed in formalin for histopathological analyses, respectively. All diagnoses were initially performed by board-certified pathologists in the context of the routine activities of veterinary pathology laboratories and were subsequently peer-reviewed and confirmed by a board-certified pathologist (JA). To fully confirm the histiocytic nature of the neoplastic cells, immunohistochemical expression of ionized calcium-binding adapter molecule (Iba1), a specific marker of the monocyte/macrophage lineage, was demonstrated in all samples from all affected dogs [13]. The age at diagnosis was calculated as the difference between the date of histological or cytological diagnosis and the date of birth.

Two histiocytic sarcoma cell lines from the sample 5472 were developed from histiocytic sarcomas tumors isolated from the lung and spleen of the same dog as previously described) [14]. These cell lines were cultured in RPMI 1640 medium (Gibco) supplemented with 10% fetal bovine serum and 0.2% primocin.

### 2.2 DNA extraction

Samples were treated as previously described [15]. Briefly, tumor DNA was extracted from RNAlater preserved tumor samples or cell lines with DNA Nucleospin Tissue kits while germline DNA was isolated from blood using the Nucleospin Blood L kit (Macherey Nagel, Düren, Germany), according to the manufacturer protocol.

### 2.3 Identification of *PTPN11* mutations by ddPCR

Known hotspot mutations in the *PTPN11* gene are named according to the international convention for point mutations, which specifies the original amino acid, the residue position in the protein, and the mutated amino acid. The nomenclature of the known mutations is provided in S1 Table and the primers are presented in S2 Table. The identification of *PTPN11* mutations in different tumor samples was performed using using ddPCR as described in Prouteau et al. (2021). Briefly, a 20 μL reaction volume was prepared containing the droplet Supermix (Bio-Rad Laboratories, Hercules, CA, USA) with a final concentration of 500 nM of forward and reverse primers, 250 nM of HEX- and FAM-labeled probes, and 10 ng of DNA. Next, the PCR reaction mixtures were partitioned into an emulsion of approximately 20,000 droplets using a QX200 ddPCR droplet generation system (Bio-Rad). PCR was conducted using the GeneAmp PCR system 9700 thermocycler (Applied Biosystems) using the following program: 95 °C for 10 min; 40 cycles of 94 °C for 30 s, and an elongation temperature of 58°C for 60 s, and subsequently 98 °C for 10 min. The ddPCR assay included corresponding positive samples from tumours in which the mutations had previously been identified by Sanger sequencing [4], matched blood samples from the same individuals, and a blank control (water). Serial dilutions of positive DNAs in normal wild type DNA, were used to determine the sensitivity of our ddPCR assay for detecting E76K and G503V mutations in the PTPN11 gene in 10ng of DNA input. These sensitivities are 0.096% and 0.2% for hotspots 1 (E76K) and 2 (G503V), respectively.

### 2.4 WGS sequencing of the 2 HS cell lines and somatic alteration identification

Whole genome sequencing (WGS) of the two HS cell lines was performed using the BGI sequencing platform (BGI, Shenzhen, China), using BGISEQ-500 short-read sequencing as previously described (Anais Prouteau et al. 2022.). Briefly, 1 µg of genomic DNA was quantified using a Qubit 3.0 fluorometer (Life Technologies, Paisley, UK), and was sheared using an E220 Covaris instrument (Covaris Inc., Woburn, MA, USA). Size selection was subsequently performed using the Vahstm DNA Clean beads kit (Vazyme, Nanjing, China) to obtain fragments with an average size of 200–400 bp. The selected fragments were end repaired and 3’ adenylated, and BGISEQ-500 platform-specific adaptors were ligated to the A-tailed fragments. The ligated fragments were purified and then amplified using PCR. Finally, circularization was performed to generate single-stranded DNA circles. After quantification, the libraries were loaded onto a sequencing flow cell and processed for 100 bp paired-end sequencing on the BGISEQ-500 platform.

After sequencing, the raw reads were filtered (adapter sequences, contamination, and low-quality reads were removed) according to the manufacturer’s guidelines. Sequence data were then aligned to the dog reference genome (assembly version canFam3.1) using BWA-MEM (version 0.7.17) [16], and PCR duplicate reads were removed using Picard tools (version 2.18.23) (http://broadinstitute.github.io/picard/, accessed on 1 July 2021). The read data were processed according to the GATK best practices; specifically, the base quality score recalibration was assessed using GATK4 (version 4.0.12).

The Mutect2 tool from the GATK4 software was then used to call somatic SNVs and short INDELS against a panel of normal (PON), comprising matched normal sample, and against germline variants from 722 dog genomes (https://data.broadinstitute.org/vgb/Ostrander_VCFs/722g.990.SNP.INDEL.chrAll.vcf.gz), accessed on 1 July 2021). Variant annotation was performed using the VEP program [16] with the EnsEMBL “Canis familiaris” annotation (v. 95). To confirm the common origin of the two HS cell lines, HaplotypeCaller tool from GATK4 software was used to call the germinal variants overlapping the 170K SNPs of Illumina CanineHD BeadChip. The identity-by-state (IBS) matrix was computed using the snpgdsIBS function of the SNPRelate R package, and a hierarchical clustering (average linkage) was then performed on the IBS-based distance matrix for these cell lines and germline DNA from 750 published BMDs [17].

### 2.5 Low pass sequencing

To confirm the tumoral status of the samples and to demonstrate that detection of divergent clones using the somatic mutational status of *PTPN11* can be missed in wild-type organs, we performed low-pass sequencing on tumor samples. These included one case (dog 8074), which exhibited a *PTPN11* mutation in the lung but had a wild-type status in the spleen and liver, one case with wild-type tumor pairs (dog 17092), and one case with mutated tumor pairs (dog 18716). Low-pass sequencing of tumor samples was performed by Psomagen (Rockville, MD, USA) as previously described (Anais Prouteau et al. 2022.). Briefly, the input DNA quality was verified using gel electrophoresis, and the quantity was measured using the Picogreen assay (Thermo Scientific, Waltham, MA, USA); 2 ng of DNA was prepared in 30 ul of buffer and used for library construction using the Nextera DNA Flex Library Kit (Illumina, San Diego, CA, USA), according to the manufacturer’s guidelines. The size of the final DNA libraries was then validated using the TapeStation D1000 ScreenTape (Agilent, Santa Clara, CA, USA) and D1000 reagents (Agilent, Santa Clara, CA, USA). The quantity was measured using the Picogreen assay (Thermo Scientific, Waltham, MA, USA), and the molar concentration was calculated using both sources. The libraries were normalized to 2 nM, pooled in equimolar volume, and then loaded on the flow cell from the Novaseq S4 300 cycle kit (Illumina, San Diego, CA, USA) and the XP-4lane kit (Illumina, San Diego, CA, USA). The prepared flow cell and SBS cartridge from the Novaseq S4 300 cycle kit (Illumina, San Diego, CA, USA) were inserted into the Novaseq 6000 system and sequenced using 151-10-10-151 running parameters, reaching a mean depth of 0.48 X.

After sequencing, the raw reads were processed as for the WGS (see paragraph 2.4). The somatic copy number variants (CNVs) were determined using the “R” Package DNAcopy [18]. The canine genome was split into bins with a window size of 100 kb. The number of reads was normalized to the total number of reads per sample. The log2 ratio with the corresponding germinal DNA, when available (or with a pool of normal DNAs), was estimated for each window. Several windows were merged (segmentation) into a larger segment with a significance threshold of 1 × 10^4^, and the copy number for each segment was estimated by converting the segment’s average log2 ratio into an absolute copy number, where a log2 ratio of 0 corresponds to a normal diploid state.

## 3 Results

### 3.1 Cohort characteristics

Thirty-eight individuals with abdominal and thoracic HS were collected between 2005 and 2019 and included in this study (Table 1). This cohort include 34 Bernese Mountains Dogs (BMDs; 89.5%), 3 Rottweilers (Rott; 7.9%) and one Flat Coated Retriever (FCR; 2.6%). This proportion reflects the described predisposition of BMDs to DSH. No sex bias was observed in this cohort with 21 (55.3%) males and 17 (44.7%) females (Chi-square test, p = 0.51). The median age of HS at diagnosis in this population was 7.09 years old (range: 2.23–11.73 years).

**Table 1:** Genotyping of *PTPN11* mutations in the cohort of 38 dogs.

ID	Breed	Sex	Age	Organ	Mutation E76	Mutation G503	Conclusion
2287	BMD	F	6.42	Lung	WT	G503V (AF 60.8%)	Discordant_mutations_between organs
				Liver	E76K (AF 55.3%)	WT	
5472	BMD	F	7.9	Lung	E76K (AF 45.4%)	WT	Discordant_mutations_between organs
				Spleen	WT	G503V (AF 50%)	
2558	BMD	F	5.78	Lung	E76K (AF 31.3%)	WT	Discordant_mutations_between organs
				Spleen	E76G (AF 10.1%)	WT	
8074	BMD	M	7.46	Lung	E76K (AF 20.1%)	WT	Discordant_mutated_vs_WT
				Spleen	WT	WT	
				Liver	WT	WT	
10273	BMD	M	7.3	Lung	WT	G503V (AF 11.8%)	Discordant_mutated_vs_WT
				Spleen	WT	WT	
8357	BMD	M	4.95	Lung	WT	WT	Discordant_mutated_vs_WT
				Spleen	E76K (AF 1.48%)	WT	
17417	BMD	M	5.31	Lung	WT	WT	Discordant_mutated_vs_WT
				Liver	E76K (AF 2.14%)	WT	
2850	BMD	M	5.25	Lung	E76K (AF 15,7%)	WT	Concordant_mutation
				Liver	E76K (AF 45.2%)	WT	
				Spleen	E76K (AF 47%)	WT	
4065	BMD	F	9.09	Lung	E76K (AF 45.3%)	WT	Concordant_mutation
				Spleen	E76K (AF 53%)	WT	
4237	BMD	M	5.18	Lung	E76K (AF 28.9%)	WT	Concordant_mutation
				Spleen	E76K (AF 55.3%)	WT	
5009	BMD	F	8.91	Lung	E76K (AF 76.6%)	WT	Concordant_mutation
				Ovary	E76K (AF 74.4%)	WT	
6746	BMD	F	6.19	Lung	E76K (AF 70.5%)	WT	Concordant_mutation
				Liver	E76K (AF 81%)	WT	
7305	BMD	F	9.18	Lung	E76K (AF 14.3%)	WT	Concordant_mutation
				Liver	E76K (AF 69.6%)	WT	
8261	BMD	F	8.04	Lung	E76K (AF 26.6%)	WT	Concordant_mutation
				Spleen	E76K (AF 26.1%)	WT	
8833	BMD	M	10.17	Lung	E76K (AF 28.7%)	WT	Concordant_mutation
				Liver	E76K (AF 25%)	WT	
10178	BMD	M	6.98	Lung	E76K (AF 27%)	WT	Concordant_mutation
				Liver	E76K (AF 51.3%)	WT	
13225	BMD	M	6.37	Lung	E76K (AF 16%)	WT	Concordant_mutation
				Liver	E76K (AF 34.5%)	WT	
18736	BMD	M	7.1	Lung	E76K (AF 2.18%)	G503V (AF 27.4%)	Concordant_mutation
				Spleen	E76K (AF 5.66%)	G503V (AF 7.95%)	
10013	BMD	F	5.66	Lung	E76K (AF 16.3%)	G503V (AF 5%)	Concordant_mutation
				Spleen	E76K (AF 29.4%)	G503V (AF 30.6%)	
8068	BMD	M	7.81	Lung	E76K (AF 42%)	WT	Concordant_mutation
				Liver	E76K (AF 1.7%)	WT	
13061	BMD	M	6	Lung	E76K (AF 3%)	WT	Concordant_mutation
				Spleen	E76K (AF 26.3%)	WT	
6727	Rott	F	6.89	Lung	E76K (AF 32.6%)	WT	Concordant_mutation
				Kidney	E76K (AF 21%)	WT	
11050	Rott	F	10.68	Lung	E76K (AF 13.1%)	WT	Concordant_mutation
				Liver	E76K (AF 21%)	WT	
6847	FCR	F	8.61	Lung	E76Q (AF 18.2%)	WT	Concordant_mutation
				Spleen	E76Q (AF 23.9%)	WT	
5206	BMD	M	4.68	Lung	WT	G503V (AF 25.7%)	Concordant_mutation
				Liver	WT	G503V (AF 10%)	
1108	BMD	M	7.08	Lung	WT	G503V (AF 70.3%)	Concordant_mutation
				Liver	WT	G503V (AF 66.8%)	
5026	Rott	F	7.29	Lung	WT	WT	Concordant_mutation
				Liver	WT	G503V (AF 20.7%)	
8505	BMD	M	7.23	Lung	WT	G503V (AF 26.6%)	Concordant_mutation
				Spleen	WT	G503V (AF 27.7%)	
8953	BMD	F	5.62	Lung	WT	G503V (AF 34.9%)	Concordant_mutation
				Spleen	WT	G503V (AF 30.3%)	
11883	BMD	F	7.74	Lung	E76K (AF 12.8%)	G503V (35.2%)	Concordant_mutation
				Liver	E76K (AF 16.2%)	G503V (18.8%)	
9635	BMD	M	6.25	Lung	WT	G503V (AF 14%)	Concordant_mutation
				Kidney	WT	G503V (AF 50,4%)	
18716	BMD	F	5.29	Lung	E76K (AF 25.1%)	WT	Concordant_mutation
				Liver	E76K (AF 49.6%)	WT	
5022	BMD	M	9.68	Lung	WT	WT	No_mutation
				Liver	WT	WT	
8073	BMD	M	5.95	Lung	WT	WT	No_mutation
				Liver	WT	WT	
9499	BMD	M	2.23	Lung	WT	WT	No_mutation
				Liver	WT	WT	
8787	BMD	F	11.73	Lung	WT	WT	No_mutation
				Liver	WT	WT	
17092	BMD	M	9.72	Lung	WT	WT	No_mutation
				Kidney	WT	WT	
8843	BMD	M	7.26	Lung	WT	WT	No_mutation
				Spleen	WT	WT	

M = Male, F = Female, BMD = Bernese Mountain Dog, Rott = Rottweiler, FCR = Flat Coated Retriever, AF = Allele Frequency.

### 3.2 Genotyping of *PTPN11* mutations

Tumor DNA samples and corresponding germline DNAs extracted from blood samples of the 38 dogs were analyzed by using droplet digital PCR (ddPCR) to detect *PTPN11* mutations at two hotspot regions: E76 (hotspot 1) and G503 (hotspot 2). No *PTPN11* mutations were identified in germline DNAs confirming the somatic origin of these mutations.

Among the **38 dogs** studied, **25 (66%)** carried at least one mutation at the main **hotspot 1** (**E76**), and **12 (32%)** had a mutation at the second **hotspot 2** (**G503**) in at least one organ (Table 1). These frequencies are consistent with previous reports of recurrent *PTPN11* hotspot mutations in canine histiocytic sarcoma, particularly in Bernese Mountain Dogs (Hédan et al. 2020; Takada et al. 2019).

**Fig 1:**
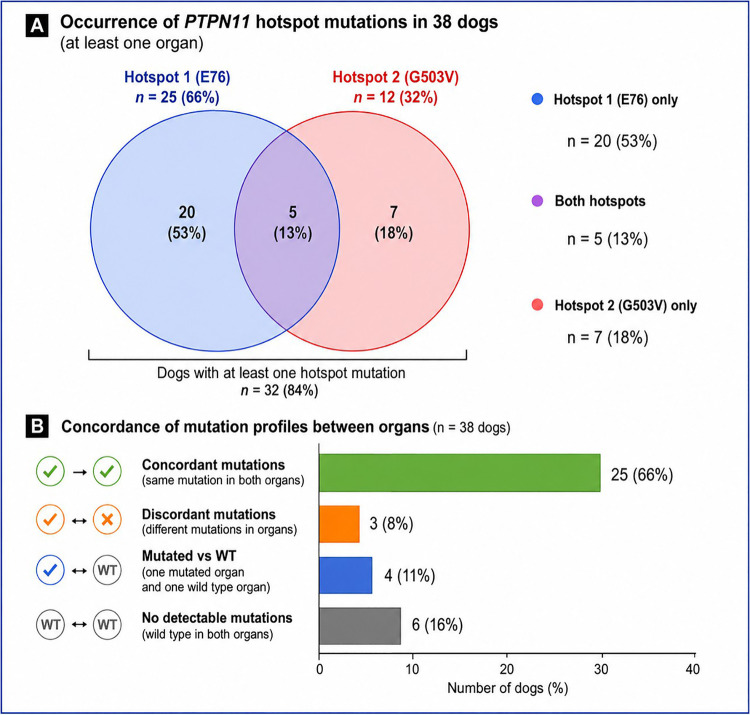
*PTPN11* mutation patterns and inter-organ concordance in 38 dogs with DHS. (A) Venn diagram showing overlap between hotspot mutations. Among 38 dogs, 25 (66%) carried an E76 mutation and 12 (32%) a G503V mutation. Five dogs (13%) harbored mutations at both hotspots, resulting in a total of 32 (84%) dogs with at least one mutation. (B) Concordance of mutation profiles between organs. Most cases (25/38, 66%) showed concordant mutations, while 3 (8%) had discordant mutations, 4 (11%) showed mutated versus wild-type profiles, and 6 (16%) had no detectable mutations.

Mutation profiles were compared between organs to assess concordance between organs. Most dogs showed concordant profiles between organs (25/38, 66%). In contrast, seven of 38 cases (18.4%) exhibited discordant profiles between abdominal and thoracic histiocytic sarcoma (HS) lesions, including three cases (7.9%) with differing mutations and four cases (11%) with mutated versus wild-type profiles. Notably, all discordant cases occurred in Bernese Mountain Dogs. The complete results are summarized in Fig 1.

### 3.3 Estimation of divergent clone frequency using somatic mutational status of *PTPN11*

As *PTPN11* is frequently mutated in HS at two main hotspots (E76 and G503), these sites can be used to assess clonal divergence between different tumors from the same dog. However, in cases where both organs were wild type, no somatic mutation was available to evaluate potential clonal divergence. In addition, for cases with a mutation in one organ and a wild-type status in the other, we could not rule out that the tumor cell fraction in the wild-type organ was too low to be detected, even with sensitive techniques such as ddPCR. To illustrate this, low-pass sequencing was performed on one case (dog 8074) presenting a mutation in one organ and a wild-type status in the other, one case with wild-type tumor pairs (dog 17092) and one case with mutated tumor pairs (dog 18716). Analysis of copy number alterations (CNAs) detected by low-pass sequencing confirmed the presence of tumor tissue in all tumor tissue samples including the wild-type samples (S1-S3 Figs). In addition, Spearman correlation analysis revealed high CNA similarity only between the mutated paired samples of dog 18716, thereby identifying divergent clonal origins in the two other dogs (S4-S5 Figs; S3 Table). These results confirm the existence of divergent clonal patterns among tumors classified as wild type by *PTPN11* hotspot analysis.

Thus, to estimate the frequency of divergent clonal patterns, we focused only on cases with different *PTPN11* mutations in the abdominal and thoracic HS lesions among cases with mutated tumor pairs, i.e., three dogs out of 28. However, only a subset of divergent situations can be detected with this approach, because the same somatic mutations are recurrent and frequent across different dogs—E76 in 30.2 to 48.6% of cases and G503 in 9–10% [4,5]. Consequently, identical mutations observed in two organs in the same dog may have arisen independently, particularly at the E76 hotspot. Thus, the frequency of HS originating from different clones, based only on cases in which distinct *PTPN11* mutations, is underestimated and must be corrected to account for situations that are not detectable with our two-hotspot based approach.

In a scenario where two HS lesions from the same dog acquire independent *PTPN11* mutations, the probability of detecting a mismatch between the two tumors (P_detected) corresponds to the probability that the lesions harbor different hotspot mutations (one at E76 and the other at G503).Let p_1_ and p_2_ denote the frequencies of mutations at hotspot 1 (E76) and hotspot 2 (G503), respectively, calculated among *PTPN11*-mutated cases. A detectable mismatch can arise only in two configurations:

1. E76 in the abdominal lesion and G503 in the thoracic lesion, or
2. G503 in the abdominal lesion and E76 in the thoracic lesion.

Each configuration occurs with probability ***p_1_ × p_2_***. Therefore, the probability of detecting a mismatch among independently mutated pairs is:


$$\begin{array}{c} P\_detected=2\times p_{1}\times p_{2} \end{array}$$

Because our current cohort is limited (32 mutated tumor pairs), the statistical estimate of p1 and p2 may be unstable. To obtain more robust estimates, we applied published *PTPN11* hotspot frequencies from a larger Bernese Mountain Dog cohort (Hédan et al. 2020; Takada et al. 2019), where p_1_ (E76) = 0.756–0.852 and p_2_ (G503) = 0.148–0.244. Using these values, the proportion of detected mismatches are:


$$\begin{array}{c} \text{P}\_\text{detected},\text{min}=\text{2}\times \text{0}.\text{756}\times \text{0}.\text{148}=\text{0}.\text{2237} \end{array}$$

$$\begin{array}{c} \text{P}\_\text{detected},\text{max}=\text{2}\times \text{0}.\text{852}\times \text{0}.\text{244}=\text{0}.\text{4158} \end{array}$$

In our cohort, 3/28 (10.7%) mutated tumor pairs showed discordant *PTPN11* mutations. Adjusting for the limited detection based on hotspot-based analysis, the estimated proportion of tumor pairs truly arising from acquire independent *PTPN11* mutations, (P_present) is:


$$\begin{array}{c} \text{P}\_\text{present},\text{min}=\text{0}.\text{107}/\text{0}.\text{4158}=\text{0}.\text{2573} \end{array}$$

$$\begin{array}{c} \text{P}\_\text{present},\text{max}=\text{0}.\text{107}/\text{0}.\text{2237}=\text{0}.\text{4789} \end{array}$$

Thus, since only a subset of potential divergent *PTPN11* mutations can be detected using the two hotspots, we estimate that in this cohort, the theoretical proportion of tumors arising from independent *PTPN11* clones could be up to three times higher than the number directly observed (10.7%).

### 3.4 Exploration of the HS clonality by analyzing cell lines from a DHS case harboring different mutations

To further investigate the distinct mutational profiles observed across organs affected by DHS, we performed whole-genome sequencing (WGS) on two cell lines derived from different tumor sites (spleen and lung), as well as on germline DNA (blood) from dog 5472, a case of disseminated HS (Table 1). Germline DNA and DNA from both tumor-derived cell lines were sequenced at an average depth of 30 × . Whole-genome copy number alteration (CNA) profiling revealed extensive genomic rearrangements in both cell lines (S6 Fig). SNPs from the Illumina 173K Canine HD array were extracted from the whole-genome sequencing data of the germline germinal DNA and the spleen and lung cell lines. Hierarchical clustering, based on a genome-wide genetic distance matrix derived from identity-by-state (IBS) similarity and calculated from SNP genotypes (including dog 5472 and 749 additional samples from the cohort [17]) using SNPRelate, confirmed that the two 5472-derived cell lines originated from the same dog 5472 (S7 Fig). Then, the Mutect2 program was used to catalog a total of 33332 and 26475 SNVs in the lung and the spleen derived cell lines, respectively (Table 2, S4-S5 Tables). Mutations in genes frequently altered in HS, such as *TP53*, or *PTPN11* [4,5,7,8], were detected in both cell lines (K128N, E169K, G256E for *TP53* and E76K, G503V for *PTPN11*). Among all identified variants, only one SNV (Chr7: 5963091 C > G) was shared between the two cells lines. This variant is a predicted deleterious coding variant in *MRPL45*, a gene encoding a mitochondrial ribosomal protein. With only one common variant – representing 0.003% and 0.0037% of variants in the lung and spleen cell lines, respectively – we can conclude that the two cell lines originated from different clones.

**Table 2:** Summary of SNVs detected in the two cell lines Dog_HS_5472_Lung and Dog_HS_5472_Spleen and identification of 1 deleterious variant common to both cell lines.

	Dog_HS_5472_Lung	Dog_HS_5472_Spleen	common to both cell lines	common to both cell lines after filtration for repeat regions
intergenic_variant	15509	12923	96	0
upstream_gene_variant	1809	1237	17	0
downstream_gene_variant	1710	1344	15	0
UTR_variant	202	104	0	0
intron_variant	13663	10600	91	0
synonymous_variant	102	57	1	0
frameshift_variant	7	11	0	0
inframe_deletion	1	1	0	0
missense_variant	179	117	1	1 (Deleterious in the gene MRPL45)
non_coding_transcript_exon_variant	85	55	1	0
splice_acceptor_variant	1	1	0	0
splice_donor_variant	2	1	0	0
splice_region	39	19	0	0
stop_gained	23	5	0	0
				
Total	33332	26475	222	1

## 4 Discussion

In disseminated histiocytic sarcoma (DHS), formerly referred to as malignant histiocytosis, multiple organs are affected at the time of diagnosis. It has generally been assumed that these tumors originate from a single clone, with early dissemination of tumor cells to distinct organs. However, in this cohort, we show that, in some dogs, thoracic and abdominal tumors display independent *PTPN11* mutation clonal statuses in at least 10.7% of cases, with the theoretical proportion potentially reaching 25–48% of cases. In addition, whole-genome sequencing of two cell lines derived from tumors in different sites of a single dog with DHS revealed that only one somatic mutation, representing 0.003% of the variants, is shared between the two cell lines. While prolonged culture could artificially introduce molecular differences through the accumulation of specific mutations in certain cell lines, common initiating mutations should still be present and detectable. This finding demonstrated that these tumors originated from clonally independent neoplastic histiocytes. These findings suggest that, in DHS, thoracic and abdominal tumors may be genetically unrelated in nearly one-third of the dogs, challenging the traditional model of clonal dissemination.

In most cancers, it is generally expected that a single primary tumor disseminates through metastatic spread, rather than multiple independent tumors of the same cell type arising within the same individual. We previously hypothesized that DHS could originate from a single initiated cell, followed by subsequent genetic divergence through the acquisition of *PTPN11* mutations associated with metastasis [4]. In this study, we identify that a substantial proportion of independent *PTPN11* mutations can arise within cells from the same individual affected by DHS. Several hypotheses could potentially explain this result. First, all cases were detected in the Bernese Mountain Dogs (BMD), a breed highly predisposed to HS, particularly the disseminated form [10,12,19,20]. This higher predisposition may be associated with an increased probability of developing independent HS tumors simultaneously. Further studies in other frequently affected dogs, such as Rottweiler and Flat-Coated Retrievers, are needed to determine whether this situation also occurs in those breeds and at what frequency. From a technical standpoint, selecting cases showing neoplastic dissemination to both the abdomen and thorax may overestimate the frequency of independent tumors. However, focusing on *PTPN11* mutations is likely to capture only a subset of tumors with distinct clonal origins. Future studies involving larger cohort and next-generation sequencing (NGS), such as low-pass or exome sequencing, are necessary to determine the exact proportion of independent tumors in DHS. In particular, low-pass sequencing enables the identification of copy number alterations (CNAs) and confirms that the *PTPN11* wild-type tumor analyzed are indeed neoplastic in nature as illustrated in this study by the low-pass analysis of the dogs 8074 and 17092. Such analyses should be extended to verify the neoplastic nature of all wild-type samples and to include in the analysis dogs presenting a mutation in one organ and a wild-type status in another. Additionally, exome analysis of common aberrations - single nucleotide variants (SNVs) and copy number alterations (CNAs) – should help to determine whether these clonal diversities result from the emergence of subclonal *PTPN11* mutations at late tumor stages, as previously suggested [4] or whether distinct tumors arise early and independently from genetically fragile histiocytic cells in the same dog, as indicated by the whole-genome sequencing analysis of dog 5472 in this study. Such studies will be also valuable for determining the chronology of mutational events during HS progression.

Altogether, these results have important implications for the management of this devastating cancer, particularly regarding targeted therapeutic strategies and the occurrence of natural treatment resistance. Current DHS treatment typically involves tumor removal (when feasible) combined with chemotherapy [21]. The presence of distinct somatic mutations among independent clones could contribute to heterogeneous therapeutic responses, especially when targeted therapies are used [22], as exemplified by dogs with *c-KIT*–mutated mast cell tumors, which exhibited objective response rates to tyrosine kinase inhibitors that were twice as high as those of non-mutated cases [23]. Finally, research on DHS therapies should account for the presence of independent tumors and determine whether cancer relapse results from pre-existing original clones that evade therapy, or from the emergence of an independent clone, in order to accurately evaluate treatment efficacy.

Such heterogeneity of somatic mutations in multicentric tumors has been rarely, but previously, described in canine mast cell tumors or mammary tumors [24,25]. Since this observation is likely linked to the strong breed predisposition for HS in dogs, it is not expected to occur at a similar frequency in the rare human HS cases. However, comparable situations have already been reported in human cancers, including gastrointestinal stromal tumors, where polyclonal evolution with intratumoral *KIT* mutational heterogeneity has been observed [26], colorectal cancer, where a polyclonal-to-monoclonal transition has been documented [27] or in multifocal ileal neuroendocrine tumors [28]. More broadly, multiclonality has been documented across all tumor stages and within 53 different types of human tumors [29]. Several non-exclusive mechanisms have been proposed to explain multiclonality in tumors: (i) predisposing genetic factors, (ii) the field cancerization, driven by environmental carcinogens or cancer-predisposing diseases (e.g., ulcerative colitis, Crohn’s disease, and Barrett’s esophagus), which create a mutagenic microenvironment favoring independent clonal expansion. In addition, (iii) clonal recruitment and cooperation, observed in mouse, which facilitates tumor transformation and may explain multiclonality in many tumors [29].

## 5 Conclusion

By studying *PTPN11* mutations in disseminated histiocytic sarcoma affecting organs located in different body compartments (thorax and abdomen), we estimate that in Bernese Mountain Dogs a significant proportion of tumors present a clonal diversity and independent clonal origin of *PTPN11* mutations. Our results suggest that many affected dogs may develop several clonally distinct histiocytic tumors either simultaneously or within a short period of time. Given the major implications of this frequent clonal diversity for the therapeutic management of this aggressive cancer, this genetic feature of canine oncogenesis warrants further investigation, particularly from a comparative perspective aimed at improving the treatment of DHS in both dogs and in humans.

## Supporting information

S1 Table Nomenclature of known mutations for *PTPN11.* (XLSX)

S2 Table Primers used in the study for *PTPN11* mutation detection, using ddPCR methods. (XLSX)

S3 Table CNAs detected on low-pass sequencing of the 3 tumors of the dog 8074. (XLSX)

S4 Table SNVs detected by whole genome sequencing in the cell line Dog_HS_5472_Lung. (XLSX)

S5 Table SNVs detected by whole genome sequencing in the cell line Dog_HS_5472_Spleen. (XLSX)

S1 Fig Low-pass whole-genome CNAs profiles detected in the three tumors of dog 8074. This analysis confirmed the presence of tumor tissue in both the spleen and the liver *PTPN11*-WT samples. Furthermore, spleen and liver tumors share common CNAs and breakpoints, but only one alteration- the chromosome 20 deletion- (See also S3 Table), was observed in all three tumor sites including the lung tumor suggesting that the lung tumor originated from a different (sub)clone.
(PDF)

S2 Fig Low-pass whole-genome CNAs profiles detected in the two tumors of dog 17092. This analysis confirmed the presence of tumor tissue in the two *PTPN11*-WT tumors.
(PDF)

S3 Fig Low-pass whole-genome CNAs profiles detected in the two tumors of dog 18716. This analysis confirmed that the both *PTPN11*mutated tumors share common CNAs and breakpoints, suggesting that the two tumors originated from the same clone.
(PDF)

S4 Fig Spearman correlations of the log2 read depth ratios of 8074 dog tumors. Spearman correlations analysis demonstrated significant similarity in CNAs between the spleen and liver tumors, but not with the lung tumor, supporting the hypothesis that the *PTPN11* mutated lung tumor originated from a different (sub)clone.
(PNG)

S5 Fig Spearman correlations of the log2 read depth ratios of tumors from dogs 17092 and 18716 showing a different origin between the lung and kidney tumors from the dog 17092 while lung and liver tumors from dog 18716 appear to have a common origine. (PNG)

S6 Fig Whole-genome CNAs profiles detected in the two cell lines derived from tumors of dog 5472. (PDF)

S7 Fig Clustering based on an identity-by-state (IBS) distance matrix using 173,000 SNP genotypes from 750 Bernese Mountain Dogs (BMDs), as well as samples from dog 5472 (including a spleen-derived cell line, a lung-derived cell line, and blood). The zoomed-in view confirms the shared origin of the two 5472-derived cell lines.
(PDF)
