# Supplementary figures and images for: Identification of divergent PTPN11 mutations in canine histiocytic sarcomas reveals evidence of an independent clonal origin
Source: PLoS One. 2026 Jul 20;21(7):e0345429. doi: 10.1371/journal.pone.0345429 (PMC13384320; doi:10.1371/journal.pone.0345429)

Dog\_8074\_liver

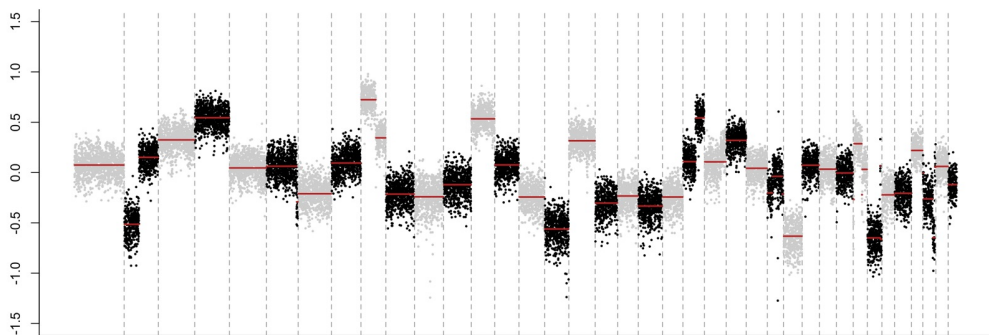

Dog\_8074\_spleen

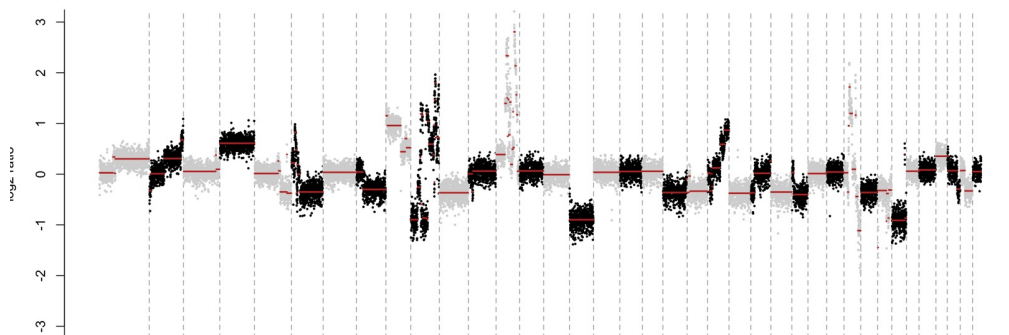

Dog\_8074\_lung

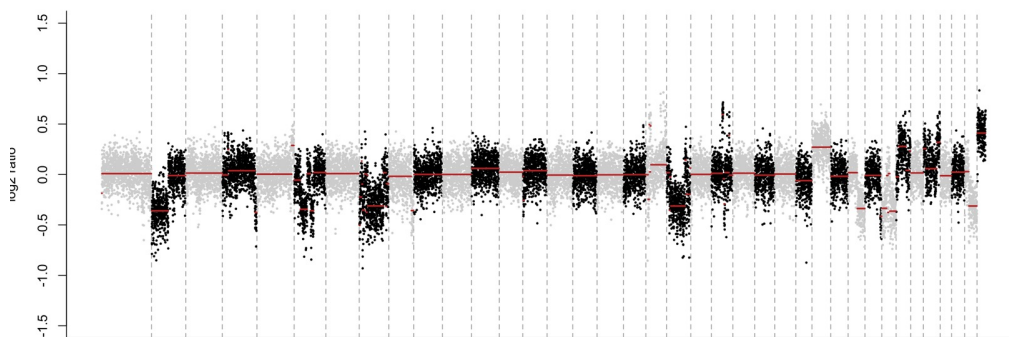

1 2 3 4 5 6 7 8 9 10 11 12 13 14 15 16 17 18 19 20 21 22 23 24 26 28 30 32 34 36 38

chromosomes

Supplement: S1 Fig — This analysis confirmed the presence of tumor tissue in both the spleen and the liver PTPN11-WT samples. Furthermore, spleen and liver tumors share common CNAs and breakpoints, but only one alteration- the chromosome 20 deletion- (See also S3 Table), was observed in all three tumor sites including the lung tumor suggesting that the lung tumor originated from a different (sub)clone. (PDF) [file pone.0345429.s006.pdf]

Dog\_17092\_kidney

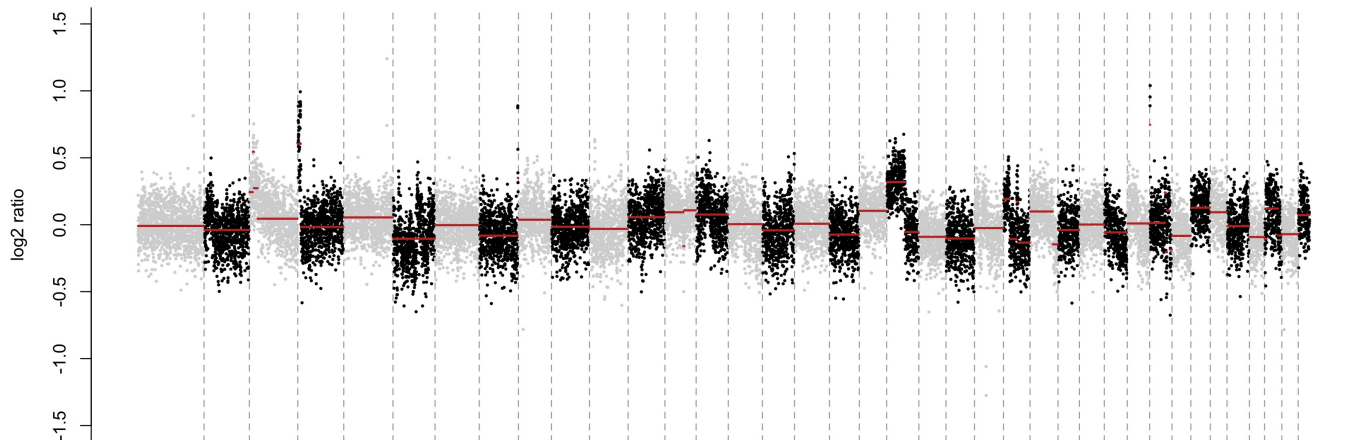

Dog\_17092\_lung

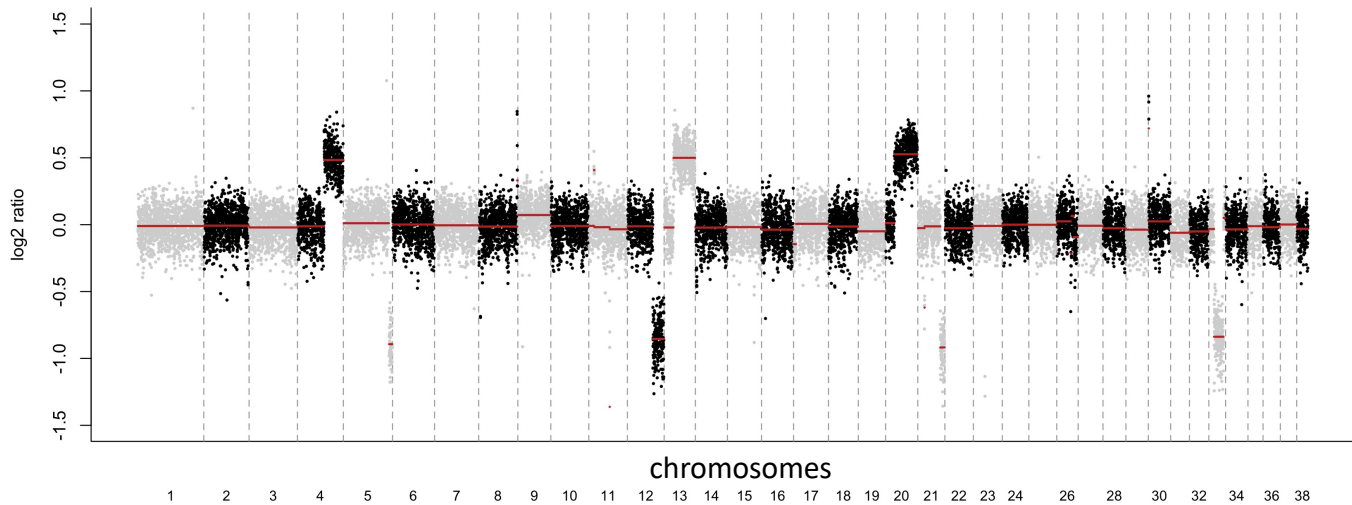

Supplement: S2 Fig — This analysis confirmed the presence of tumor tissue in the two PTPN11-WT tumors. (PDF) [file pone.0345429.s007.pdf]

Dog\_18716\_liver

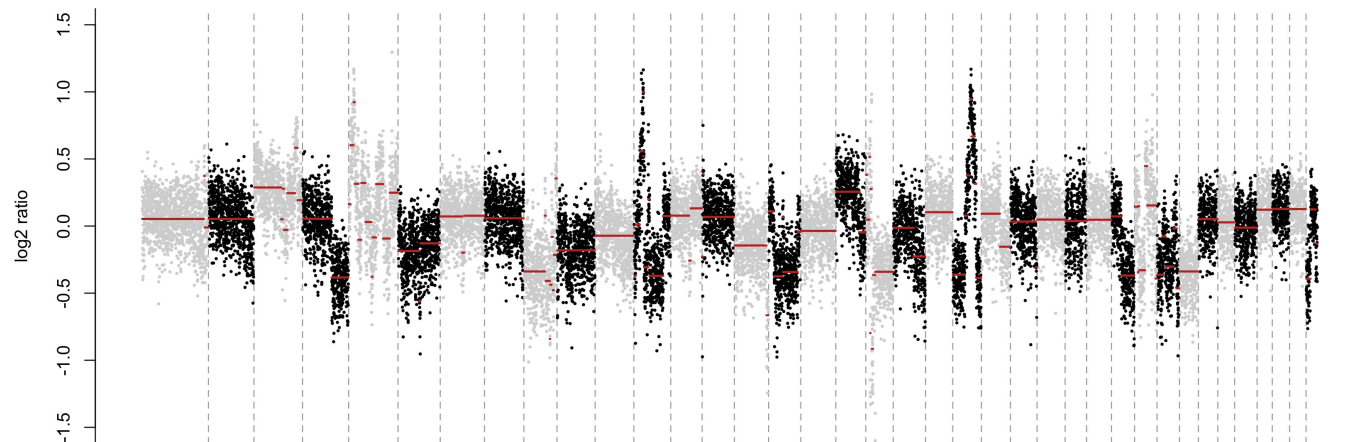

Dog\_18716\_lung

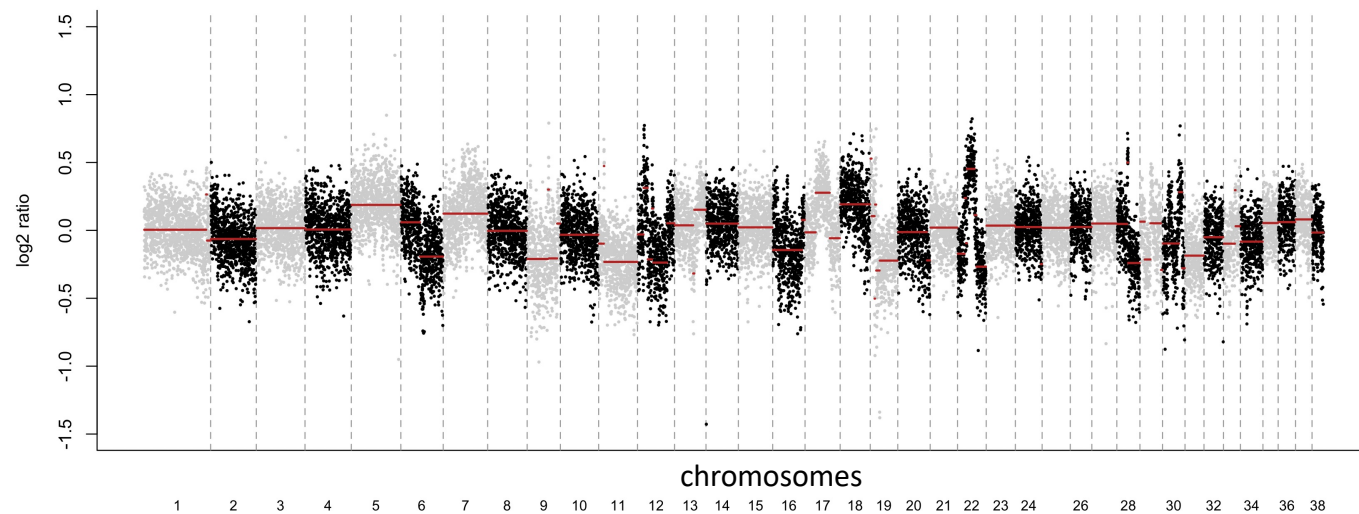

Supplement: S3 Fig — This analysis confirmed that the both PTPN11mutated tumors share common CNAs and breakpoints, suggesting that the two tumors originated from the same clone. (PDF) [file pone.0345429.s008.pdf]

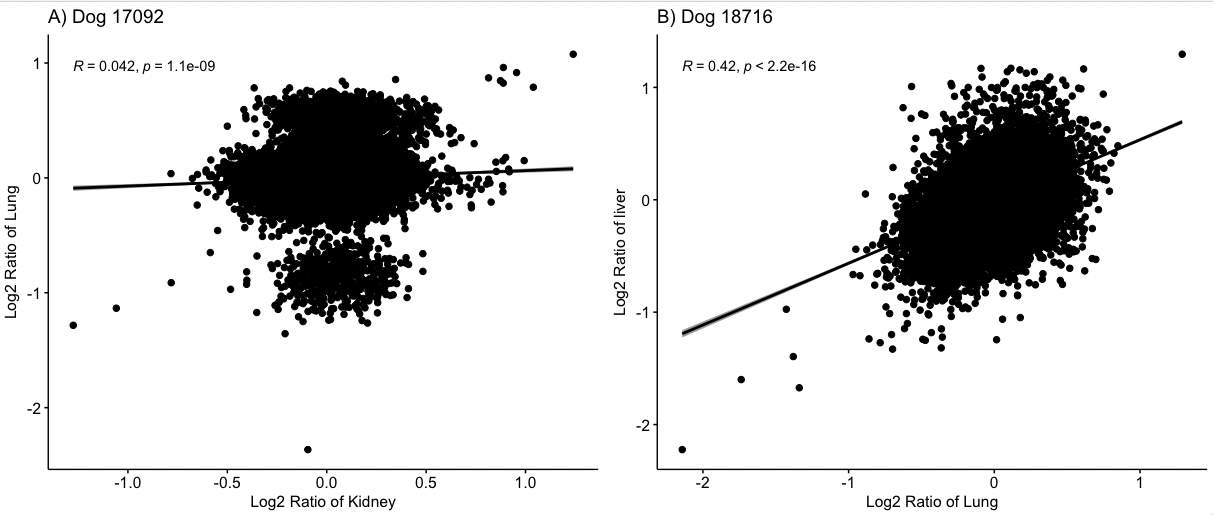

Supplement: S4 Fig — Spearman correlations analysis demonstrated significant similarity in CNAs between the spleen and liver tumors, but not with the lung tumor, supporting the hypothesis that the PTPN11 mutated lung tumor originated from a different (sub)clone. (PNG) [file pone.0345429.s009.png]

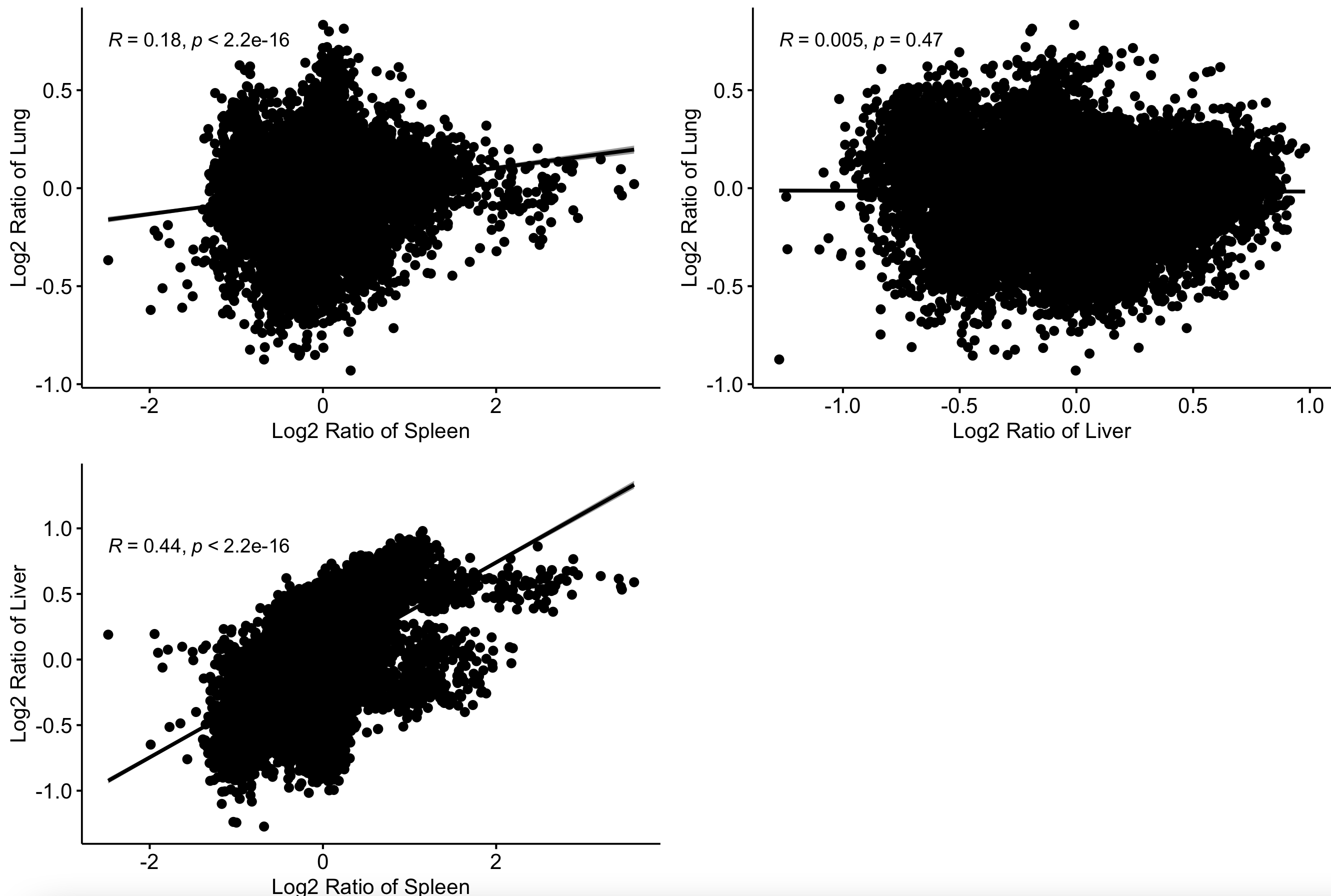

Supplement: S5 Fig — (PNG) [file pone.0345429.s010.png]

Cell\_line\_5472\_Lung

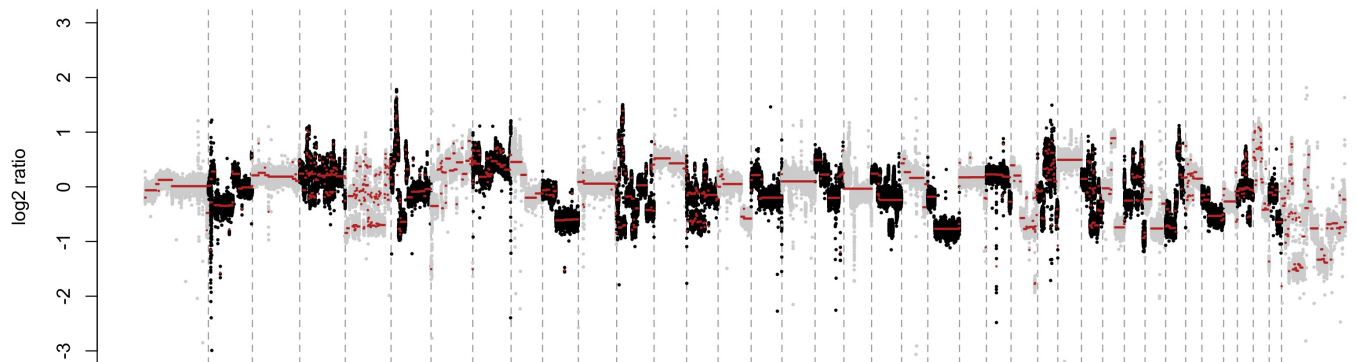

Cell\_line\_5472\_Spleen

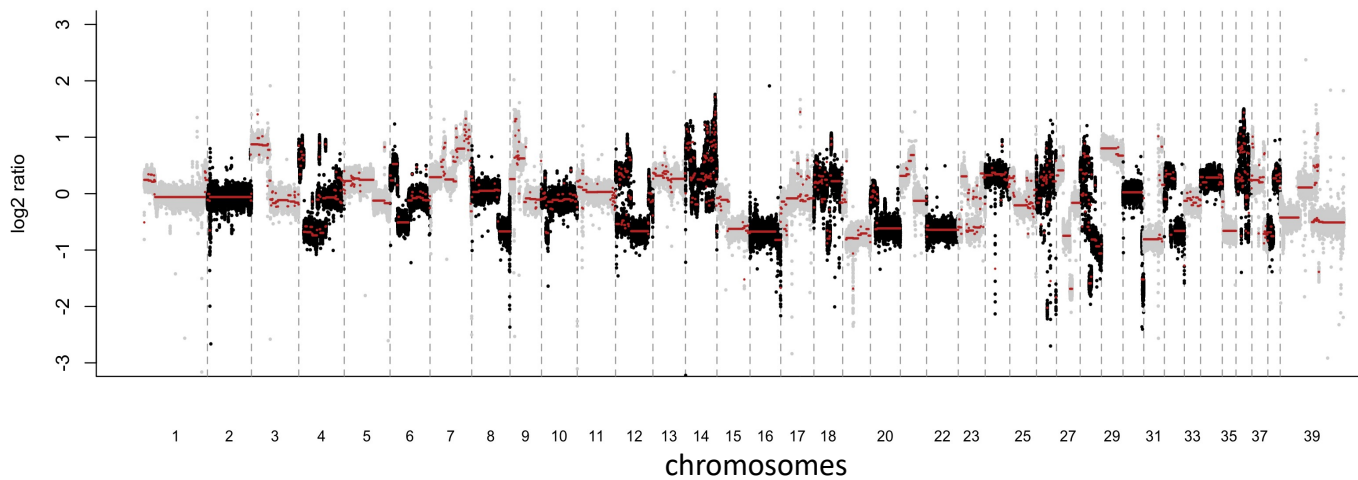

Supplement: S6 Fig — (PDF) [file pone.0345429.s011.pdf]

0 0.5 1 1.5 2

Clustering Hierarchical based on 14 distance 485

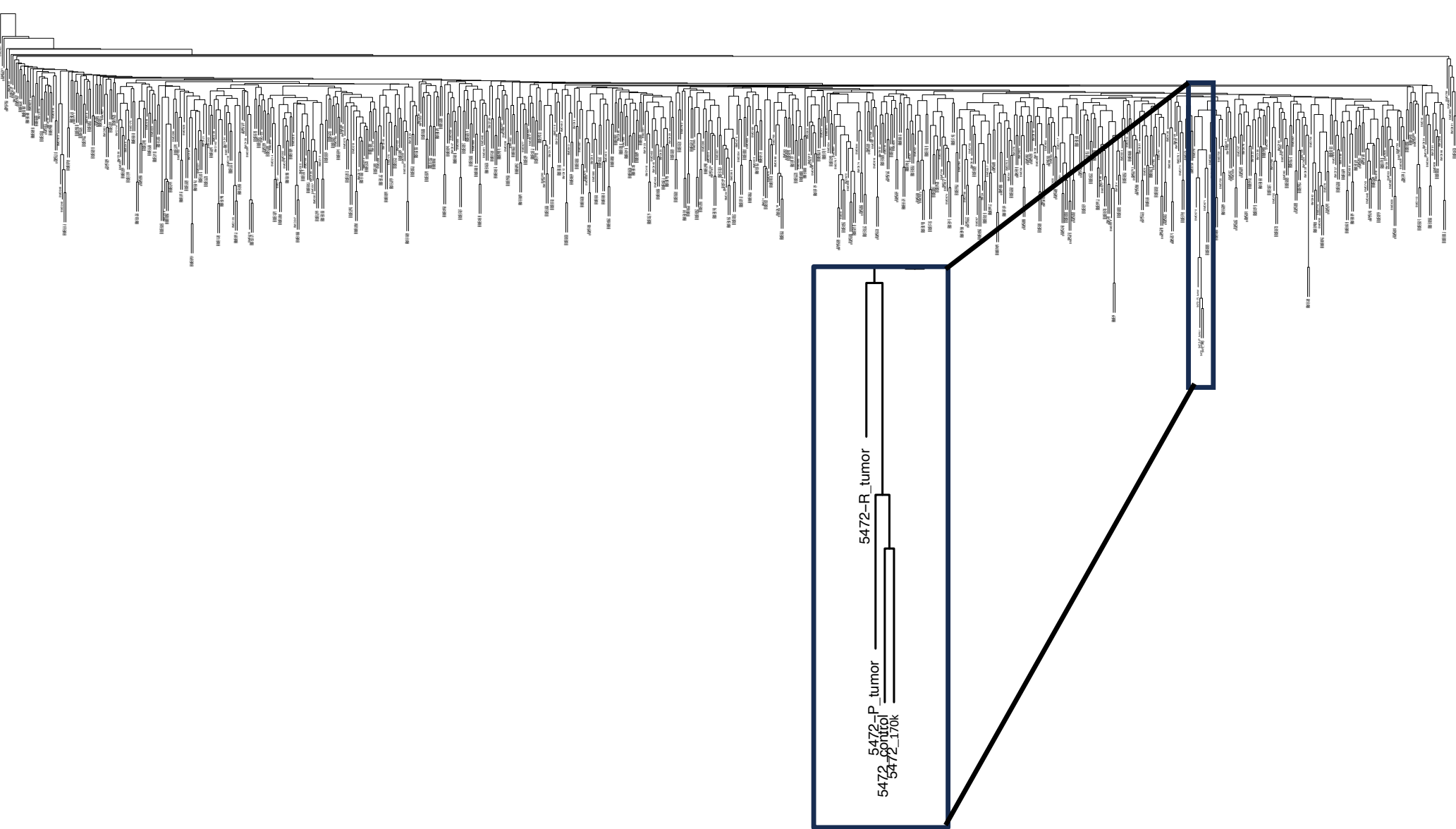

Supplement: S7 Fig — The zoomed-in view confirms the shared origin of the two 5472-derived cell lines. (PDF) [file pone.0345429.s012.pdf]
