# Supplementary material for: Identification of divergent PTPN11 mutations in canine histiocytic sarcomas reveals evidence of an independent clonal origin
Source: PLoS One. 2026 Jul 20;21(7):e0345429. doi: 10.1371/journal.pone.0345429 (PMC13384320; doi:10.1371/journal.pone.0345429)
Supplement: Attachment — Submitted filename: Answer to reviewers for the article Mottier et al.docx [file pone.0345429.s014.docx]

**Answer to reviewers for the article Mottier et al «  Investigation of disseminated canine histiocytic sarcoma reveals a polyclonal**

**origin in over 24% of cases»**

Dear Editor Douglas H. Thamm,

Thank you for reviewing this article and inviting us to submit a revised version. We would also like to thank the two reviewers for their relevant comments. We have taken in account their comments as well as your requirements. We have clarified all points in the article and addressed their comments in the point-by-point response below. The main change made to the method, in response to reviewer 1’s comments, and in order to clarify and simplify the strategy and the text, was to re-analyse all samples ( tumors and bloods samples) using ddPCR method- the most sensitive method available-, and to remove the Sanger sequencing step.

**Reviewer #1 (Reviewer Comments to the Author)**

The manuscript is very well written, and is broadly concise without lacking in detail; however, there are a few elements that are overly brief (e.g. see comments below on low-pass sequencing data).
The concept is interesting and has some level of novelty in the approach used, but largely incorporates well established techniques and principles. The subject of this study is pertinent given that canine DHS is highly aggressive and notoriously challenging to treat effectively, and shows a particularly high incidence of genomic disruption compared to many other dog cancers. It is also remarkably prevalent in the Bernese mountain dog, which allows for targeted studies that reduce the ‘noise’ associated with heterogenous genetic backgrounds. It would seem highly logical that a polyclonal origin, coupled with a propensity for continued molecular evolution, would confound the ability to control the proliferation of distinct masses within an individual. In turn, the rarity of HS in people means that there is a critical need for appropriate model systems with which to elucidate the genomic component of the disease.
The focus on PTPN11 is logical given its role in upregulating the MAPK pathway in HS of both species, and the conservation of sequence mutations at equivalent hotspots in both canine and human tumors.

My primary concern relates to conclusions being based on the extrapolation of data from 3 of 38 dogs with divergent mutations of a gene in tumors from different anatomic sites. (Note that the ‘Methods’ section of the Abstract mentions that 38 dogs were studied, but the ‘Results’ section of the Abstract implies 28). From this, the authors estimate that 24-45% of disseminated HS cases in dogs may represent polyclonal disease at the time of diagnosis, which is a very broad range. While they provide the statistical process from which they drew this conclusion, it seems somewhat preliminary at this point. The Discussion does reflect the limitations of the study, but the Abstract reads more emphatically, and the title even more so.

It is true that these results are preliminary and need to be confirmed. It is important to remain cautious about the frequency of this phenomenon, even though the estimates were calculated by applying the appropriate corrections to the usable samples (i.e., the 28 *PTPN11* mutated samples). We have therefore amended the title as suggested, as well as the abstract. The Title becomes:” Investigation of disseminated canine histiocytic sarcoma reveals an independent clonal origin in a significant number of cases”

It was unclear to me how non-neoplastic samples, mentioned in the Methods, were involved in this study. Was a germline sample from each dog screened with Sanger sequencing/digital PCR alongside the tumor samples from the same individuals?

We agree that it should be clarified that non-neoplastic samples were used as germline reference samples to confirm the somatic origin of the *PTPN11* mutations. We analysed them using Sanger sequencing, and all were tested negative. Following the reviewers’ suggestions regarding detection techniques (see below), we finally decided to test all these germline DNA samples by the ddPCR technique, they were all “*PTN11* wild type” negative and we added sentences in the results (lines 254-257, in marked-up copy of the manuscript).

I found it difficult to follow the process for evaluating the two PTPN11 mutation hotspots. For example, in Table 1:
- lung tissue for dog 10013 is shown as mutated in hotspot 1 using Sanger sequencing, but the same sample is shown as wild-type for that codon by digital PCR

This was an error, we apologize and corrected the table1. The lung tissue from dog 10013 was not tested for hotspot 1 by ddPCR, as a mutation had already been detected by Sanger sequencing. To clarify the stratey and simplify the text, we analysed all samples ( tumors and bloods samples) only by the sensitive ddPCR method, and removed the Sanger sequencing step.

During this process we detected new positive samples corrected the Table 1 accordingly.

- a lung sample from dog 2287 was wild-type for hotspot 1, and mutated in hotspot 2. In contrast, a liver sample from the same dog was mutated in hotspot 1 and wild-type in hotspot 2.
Dog 2287 was therefore classified as discordant with different mutations, but these results are only shown for the Sanger sequencing. Does this mean that neither of these samples were subsequently screened using digital PCR? Is it possible that both sites harbored both mutations, but with dissimilar allele frequencies such that only one was detectable with Sanger sequencing? Since this is the main focus of the study, I wondered why all samples were not analyzed with the more sensitive technique (i.e. digital PCR)?

You are correct; we did not perform confirmatory ddPCR testing on samples that were tested positive by Sanger sequencing, since Sanger sequencing is less sensitive than ddPCR and a sample positive by Sanger sequencing has a minor allele frequency (MAF) > 0.1-0.2. As initially described in the Materials and Methods section, the identification of *PTPN11* gene mutations in various tumor samples was first performed by Sanger sequencing. Subsequently, to improve the sensitivity of the *PTPN11* mutations detection (E76K and G503V), we re-analysed Sanger negative samples by ddPCR. Despite the limited sensitivity of Sanger sequencing, this method has the advantage of identifying mutations without prior knowledge, such as E69 in dogs 6847 and 8843, contrary to ddPCR, which targets known mutations.

However, to answer your concern and simplify the reading, we performed the dddPCR on all samples on the 2 hotspots. This analysis revealed that some samples previously classified as wild-type by Sanger sequencing did, in fact, harbor low-frequency mutations. For example, we identified two additional discordant cases initially considered wild-type: samples 8357 and 17417, which carried the E76K mutation at low allele frequencies of 1.48% and 2.14%, respectively (Please See Table1). To simplify the understanding of the manuscript, we presented only the results obtained by ddPCR and removed the Sanger sequencing results. We then modified the methods (lines 145-150), the results (lines 254-257), and Table 1, the S2 Table, the abstract (line 36), introduction (line 96).

In turn there is no mention of the relative level of sensitivity achieved by mutation screening. The sensitivity of Sanger sequencing is generally considered to be around 10-20%, but digital PCR is considerably more sensitive (depending on the quantity of input DNA and the criteria for classification of mutant vs. wild-type), and it should be possible to provide some indication of the lower limit that can be detected. Were suitable control samples run?

You are correct: the sensitivity of Sanger sequencing ranges from 10% to 20%, whereas the ddPCR assay provides accurate estimates of fractional abundance down to 0.0005% for the BRAF V600E mutation (https://pmc.ncbi.nlm.nih.gov/articles/PMC6642310/). To define the sensitivity of our ddPCR assay, we determined the limit of detection by diluting the positive control DNA in a wild type DNA, the limit of detection for hotspot 1 and 2 were 0.096% and 0.2%. Regarding to the control samples, each ddPCR run was performed in the presence of the corresponding positive mutated samples and wild-type samples, as well as a blank control (water). We added sentences regarding the ddPCR method, its sensitivity as well as these controls in the “Materials and Methods” section (lines 152-163).

The data derived from whole genome sequencing of two cells lines from one dog is well presented and compelling. It would be very useful for this approach to be carried out in the future for other individuals in the study. There is, however, no direct mention of the low-pass sequencing data (and associated figures and tables) in the Results section - the section around line 311 should be moved to the Results.

We agree that NGS approaches such as whole genome sequencing or exome sequencing should be carried out in the future, indeed we mentioned this in the discussion (lines 453-455). As suggested, we moved the low pass sequencing data in the results at the beginning of chapter 3.3 (lines 332-341).

There is very little discussion of how the findings of this study relate to the existing literature, and this should be expanded. There are several prior studies of other human and canine cancers that involve molecular comparison of multiple tumors from the same individual. Can the authors provide any insight into why an individual might develop multiple, clonally distinct lesions of the same cancer type within a short time period? Is there any precedent for this in other cancers/species? Does it point towards any particular etiology?

As suggested, we expand the discussion section, by presenting information of other studies on cancer in humans and dogs and by proposing few hypotheses to explain this situation (lines 480, 487-513).

Specific comments:

Abstract/Introduction - there is no mention of the NGS/CNV component of the study, if space allows it would be useful to include a brief reference to this, as there is currently no lead-in (first mention is line 155, with no context given)
We agree and added sentence lines 44-46 in the abstract and lines 100-102 in the introduction.

Line 92 - indicates collection of blood samples, but there is no mention of subsequent DNA extraction, and their number and use is not clear.

We agree and added the information in the material and methods (line 142) and in the results (line 254).

Line 94 refers to ‘mirror samples’, please clarify what is meant by this term.
Were any precautions taken to ensure that tumor samples were free of cross-contamination during tissue processing, and that there was minimal normal tissue in the sample? This is important when considering low abundance sequence mutations.

Mirror samples refer to a macroscopic tumour divided into two parts: one preserved in RNAlater for genomic experiments, and the other in formalin for histological analysis. Since this step is performed by the treating veterinarian, we have no control over the process. Therefore, it is critical to use highly sensitive techniques, such as ddPCR, to detect low-abundance sequence mutations. This has been clarified in the Materials and Methods section (Lines 118-119).

Line 95 - given the diagnostic challenges associated with canine HS, please provide a brief summary of diagnostic criteria. Was there any histological evidence that different tumors from the same individual were not of the same origin?

All the cases included in this study originated from the routine diagnostic activities of French veterinary pathology laboratories that are partners and contributors to our research program. The initial diagnoses were made by specialized veterinary pathologists, either European or American board-certified. For inclusion in the present project, all samples were reviewed by an ECVP diplomate pathologist (JA), who has been working for 15 years on our research program dedicated to canine histiocytic sarcomas.

The histological criteria used to diagnose a sample as histiocytic sarcoma are:

- Diffuse, infiltrative proliferation of large neoplastic round to polygonal cells
- Marked cellular pleomorphism (anisocytosis, anisokaryosis)
- Abundant pale to eosinophilic cytoplasm
- Round to oval nuclei, often with prominent nucleoli
- High mitotic index (frequent mitoses, sometimes atypical)
- Tissue destruction with effacement of normal architecture
- Frequent necrosis and/or inflammation
- Possible hemophagocytosis (in some cases)

All cases, including the different tumor samples from the same individual, fulfilled these diagnostic criteria.

As a standard procedure in all our projects, and even when the initial and secondary histopathological evaluations were concordant and consistent with a diagnosis of histiocytic sarcoma, immunohistochemical detection of the histiocytic marker Iba1 (ionized calcium-binding adapter molecule 1) was performed as a confirmatory step. We precise this in the material and methods (lines 120-126). There as not histological evidence that different tumors from the same individual were not of the same origin.

Line 98 - is it possible that the molecular divergence of these cell lines was an artifact of culture?

We agree that prolonged culture could artificially create molecular differences through the accumulation of specific mutations in certain cell lines. However, common initiating mutations should still be present and detectable, as is the case with the *PTPN11* gene mutations that have been identified. Given that there is only a single mutation between the two cell lines, we can clearly conclude that the two original tumors were independent.

Line 115 - is it possible that any samples had low frequency mutations that did not show up with Sanger sequencing, but which were not screened for with digital PCR because Sanger sequencing detected a different mutation?

We agree, and as mentioned earlier, all samples have now been analysed by ddPCR.

Line 155 - please clarify which tumor samples were analyzed with low-pass sequencing, and why these were chosen

We agree and add sentences in the result section lines 332-341.

Line 174 - this seems to be the first mention of any analysis of ‘normal’ samples, are these the blood samples mentioned in line 92?

As previously mentioned, we corrected the material and methods and the results to clarify the use of DNA from blood samples.

Line 176 - please provide more info on how the copy number of the segments was estimated

We added a sentence in the Material and Methods section, lines 238-240.

Line 186 - how was the age of onset determined?
Can the authors comment on the wide range within the cohort (age 2 to 11 years old)? Is there any significance in that?
The age of onset was calculated as the date of histological or cytological diagnosis minus the date of birth. To clarify this approach, we have added an explanatory sentence in the Materials and Methods section (lines 125-126).

Given that histiocytic sarcoma is a polyfactorial disease, the wide range in age of onset may reflect: the accumulation of predisposing mutations, as shown in Figure S6 of Hedan et al. (2021), where dogs with a higher number of risk alleles tend to have a shorter median lifespan due to an earlier onset; as well as the exposure to environmental risk factors, including chronic inflammation and the use of anti-inflammatory treatments, which have been shown to influence disease development (Ruple et al., 2016).

Lines 189-192 - this information should be moved to the Methods section.

As discussed previously, we remove all the information on Sanger sequencing, thus this information was deleted.

Following the comment on line 115, were both digital PCR assays run on samples that appeared to be wild-type for a hotspot mutation seen in a different organ?

Initially, we tested only the mutation observed in the different organs, but we have now tested all the samples at both *PTPN11* hot spots.

Lines 195-201 - this information would be more effective as a figure

We agree and added a figure (Figure 1).

Table 1 - what is the significance of the four sets of results shown in red text?

The goal was to highlight discordant results and potentially polyclonal cases. Since this information appears in the last column, the red coloured text has been removed.

Table 2 - clarify that the ‘ID’ in the second column represents the code assigned to each dog

We have removed the Table 2 since the suggested Figure 1 is clearer.

Supplementary figures - please annotate the DNA copy number information to delineate the chromosomes on the X axis, it is currently difficult to make comparisons between different samples

We delineated chromosomes as suggested.

Reviewer #2: 1. The study presents the results of original research.
2. Results reported have not been published elsewhere.
3. Experiments, statistics, and other analyses are performed to a high technical standard and are described in sufficient detail.
4. Conclusions are presented in an appropriate fashion and are supported by the data.
I would soften your conclusions in the percentage of cases with independent clones. The study indicates 3/38 dogs and the statistical model/estimate is not what you have shown.

We thank Reviewer #2 for the positive assessment and constructive feedback.

We fully acknowledge that the only three cases show a discrepancy regarding PTPN11 gene mutations, but we excluded the wild-type (WT) samples from our analysis because we cannot rule out the presence of tumor cells in these samples. We therefore excluded four potentially discordant cases (one organ with a mutation versus another of the wild-type). Furthermore, we underestimated the frequency of this phenomenon, as two tumors could have had the same mutation by chance. Low-pass analysis, allowed us to identify 2 additional discordant cases in these situations.

The 24-45% range was derived from our model, but we acknowledge that this extrapolation to the broader DHS population may appear preliminary given the limited number of observed cases. We agree and this is in line with the first comment of the first reviewer. We took in account the fact that it is important to remain cautious about the frequency of this phenomenon and we softened the language in the Abstract lines 43 and 48, in the conclusion of the Results (lines 378-381, in marked-up copy of the manuscript) and conclusion (line 520, in marked-up copy of the manuscript).

5. The article is presented in an intelligible fashion and is written in standard English.
6. The research meets all applicable standards for the ethics of experimentation and research integrity.
7. The article adheres to appropriate reporting guidelines and community standards for data availability.

Throughout the title and manuscript, rather than using the term polyclonal, independent clones or clonal diversity are more appropriate terms and used in other areas of the manuscript. This is still a neoplastic process and not reactive/inflammatory process which is typically the meaning of polyclonal. The number of clones is still very limited, not a Gaussian distribution of clones.

We agree and have made this correction in the manuscript regarding the data on histiocytic sarcoma, but not in the discussion of other cancers in humans and dogs, where the cited articles used the term “polyclonal.”

Line 20 Rather than using prognostic markers consider indicators.

We have made this correction (line 21).

Lone 88: Did you do anything with the blood from all the dogs? Looks like only the blood from the dog with cell lines was analyzed for germline mutations?

This comment is in line with a comment of the first reviewer; we have provided the necessary clarification by adding this information to the “Materials and Methods” section (line 142), and the “Results” section (line 254-257).

Line 92 Please indicate the number of dogs.

We have made this correction line 112.

Line 94 I am not sure what you mean by ‘mirror’ samples.

This comment is in line with a comment of first reviewer. This has been clarified in the Materials and Methods section (Lines 118-119).

Line 96 Pathologic diagnosis is strong when more than one pathologist reviews the case as this can be a challenging diagnosis and pathologists do not always agree. Was IHC used to confirm any cases?

All the cases included in this study originated from the routine diagnostic activities of French veterinary pathology laboratories that are partners and contributors to our research program. The initial diagnoses were made by specialized veterinary pathologists, either European or American board-certified. For inclusion in the present project, all samples were reviewed by an ECVP diplomate pathologist (JA), who has been working for 15 years on our research program dedicated to canine histiocytic sarcomas.

The histological criteria used to diagnose a sample as histiocytic sarcoma are:

- Diffuse, infiltrative proliferation of large neoplastic round to polygonal cells
- Marked cellular pleomorphism (anisocytosis, anisokaryosis)
- Abundant pale to eosinophilic cytoplasm
- Round to oval nuclei, often with prominent nucleoli
- High mitotic index (frequent mitoses, sometimes atypical)
- Tissue destruction with effacement of normal architecture
- Frequent necrosis and/or inflammation
- Possible hemophagocytosis (in some cases)

All cases, including the different tumor samples from the same individual, fulfilled these diagnostic criteria.

As a standard procedure in all our projects, and even when the initial and secondary histopathological evaluations were concordant and consistent with a diagnosis of histiocytic sarcoma, immunohistochemical detection of the histiocytic marker Iba1 (ionized calcium-binding adapter molecule 1) was performed as a confirmatory step. We precise this in the material and methods (lines 120-126).

Line 109 Section of sanger sequencing and ddPCR methods. From the references I don’t see any mention of how you have accounted for artifacts in formalin-fixed, paraffin-embedded FFPE tissue. I think it could also be useful to briefly explain the method along with the reference. One reference took me to another. The Hedan reference did not actually describe the method for sequences but instead referenced Hedan et al 2011.

All tumor samples used for genomic analyses were derived from RNAlater preserved tissue samples; therefore, there is no artifacts associated with FFPE fixation. Since we are now presenting only the results obtained using the ddPCR method, the reference Prouteau et al. 2021 is the one that refers to ddPCR methods. We clarify this point in the “Materials and Methods” section (lines 118-119) and briefly explain the method as suggested (lines 152–163).

Line 191 how many dogs had subsequent ddPCR

As mentioned, now all the samples were analysed by ddPCR. It is clarified in the Material and Methods as well the results sections (line 255).

Line 258 clonal statuses in 25-48% is theoretical or a model rather than proving this percentage in this study. I think you can say that this study has shown that anatomically different tumors display independent PTPN11 clones in 3 of 38 cases. In the discussion you could go into the estimation but I don’t believe you have displayed this in this study.

We agree with this point of view, and this is in line with the first comment of the first reviewer, saying that is preliminary and need to be confirmed in a larger cohort (what we also indicate in the discussion section). We take in account the fact that it is important to remain cautious about the frequency of this phenomenon, and we thus modified lines to not present the results in a definitive manner by softening the language in the Abstract line 48, in the conclusion of the Results (lines 378-381) and by removing this estimation in the Conclusion (line 520).
