# Supplementary material for: Identification of divergent PTPN11 mutations in canine histiocytic sarcomas reveals evidence of an independent clonal origin
Source: PLoS One. 2026 Jul 20;21(7):e0345429. doi: 10.1371/journal.pone.0345429 (PMC13384320; doi:10.1371/journal.pone.0345429)
Supplement: Attachment — Submitted filename: Answer_to_reviewers_for_the_article_Mottier_et_al_auresp_2.docx [file pone.0345429.s015.docx]

**Answer to reviewers for the article Mottier et al «  Investigation of disseminated canine histiocytic sarcoma reveals a polyclonal**

**origin in over 24% of cases»**

Dear Editor Douglas H. Thamm,

Thank you for reviewing this article and inviting us to submit a revised version. We would also like to thank the reviewers for their relevant comments. We have taken in account their comments and the remaining questions.

**Reviewer #1 (Reviewer Comments to the Author)**

The questions I raised in my review of the original submission are largely addressed in this revision. The exceptions are as follows:

In my opinion the title still reads too emphatically based on the number of samples analyzed and the proportion of tumors that show the phenomenon in question. Ultimately the data are based on observations regarding a single mutation in just three cases, with the assumption that the mutational profile of the small region of tumor sampled is representative of the whole tumor.
The use of the word ‘significant’ in the title is my key concern. I would suggest something to the effect of “Identification of divergent PTPN11 mutations in canine histiocytic sarcomas reveals evidence of an independent clonal origin”. Similarly, I would suggest modifying the running title to “Evidence of clonal diversity origin in disseminated canine histiocytic sarcoma”.

We have amended the titles as suggested.

Regarding control samples used for ddPCR analysis: If I understand correctly, the authors' comments suggest that the positive control for ddPCR was taken from among the panel of mutant-positive cases being tested for that mutation? I recommend including a statement indicating that this sample was first validated by Sanger sequencing as bearing the mutation, prior to being used as an internal positive control

We agree that it should be clarified, we corrected it lines 144-145.

Regarding the nature of blood samples mentioned: The revised text now reads “ddPCR assay included corresponding positive samples of tumors, wild-type samples, and a blank control (water)” - it is not immediately clear here that “wild type samples” refers to the patient-matched blood samples. Assuming that is the case, the term ‘wild type samples’ should be replaced by “matched blood samples from the same individuals” (or similar), since the ddPCR assay is being run to determine IF these samples are in fact wild-type.

We agree that it should be clarified, we corrected it line 145.

The term ‘mirror sample’ is not one I have encountered before, and I notice the other viewer commented the same, so I recommend the text should include the definition given in the authors' response.

You are correct the term was misleading; we have therefore removed it from the ‘Materials and Methods’ section in the latest version, whilst retaining the ‘definition’ in that same section: lines 107 to 109. “Each biopsy was divided into two parts, one was placed in RNALater solution for genomic experiments and the other was fixed in formalin for histopathological analyses, respectively. “

Regarding the possibility of molecular divergence during prolonged culture: I recommend including the authors' brief response to my query in the main text.

We added it in the discussion lines 352-356.

The term 'age of onset' should be replaced by 'age at diagnosis'

We corrected it Lines 114 and 229.
