# Supplementary material for: Identification of divergent PTPN11 mutations in canine histiocytic sarcomas reveals evidence of an independent clonal origin
Source: PLoS One. 2026 Jul 20;21(7):e0345429. doi: 10.1371/journal.pone.0345429 (PMC13384320; doi:10.1371/journal.pone.0345429)
Supplement: Peer review history

# Peer review: Decision Letter 0

**DOI:** 10.1371/journal.pone.0345429.r001

**Authors:** Douglas Thamm

5 Apr 2026

PONE-D-26-10931

Investigation of disseminated canine histiocytic sarcoma reveals a polyclonal origin in over 24% of cases.

PLOS One

Dear Dr. Hédan,

Thank you for submitting your manuscript to PLOS ONE. After careful consideration, we feel that it has merit but does not fully meet PLOS ONE’s publication criteria as it currently stands. Therefore, we invite you to submit a revised version of the manuscript that addresses the points raised during the review process.

**Please address all reviewer comments.**

Please submit your revised manuscript by May 20 2026 11:59PM. If you will need more time than this to complete your revisions, please reply to this message or contact the journal office at plosone@plos.org. When you're ready to submit your revision, log on to https://www.editorialmanager.com/pone/ and select the 'Submissions Needing Revision' folder to locate your manuscript file.

Please include the following items when submitting your revised manuscript:

- A letter that responds to each point raised by the academic editor and reviewer(s). You should upload this letter as a separate file labeled 'Response to Reviewers'.
- A marked-up copy of your manuscript that highlights changes made to the original version. You should upload this as a separate file labeled 'Revised Manuscript with Track Changes'.
- An unmarked version of your revised paper without tracked changes. You should upload this as a separate file labeled 'Manuscript'.

If you would like to make changes to your financial disclosure, please include your updated statement in your cover letter. Guidelines for resubmitting your figure files are available below the reviewer comments at the end of this letter.

If applicable, we recommend that you deposit your laboratory protocols in protocols.io to enhance the reproducibility of your results. Protocols.io assigns your protocol its own identifier (DOI) so that it can be cited independently in the future. For instructions see: https://journals.plos.org/plosone/s/submission-guidelines#loc-laboratory-protocols. Additionally, PLOS ONE offers an option for publishing peer-reviewed Lab Protocol articles, which describe protocols hosted on protocols.io. Read more information on sharing protocols at https://plos.org/protocols?utm_medium=editorial-email&utm_source=authorletters&utm_campaign=protocols.

As the corresponding author, your ORCID iD is verified in the submission system and will appear in the published article. PLOS supports the use of ORCID, and we encourage all coauthors to register for an ORCID iD and use it as well. Please encourage your coauthors to verify their ORCID iD within the submission system before final acceptance, as unverified ORCID iDs will not appear in the published article. *Only*  the individual author can complete the verification step; PLOS staff *cannot*  verify ORCID iDs on behalf of authors.

We look forward to receiving your revised manuscript.

Kind regards,

Douglas H. Thamm, V.M.D.

Academic Editor

PLOS One

Journal Requirements:

When submitting your revision, we need you to address these additional requirements.

1. Please ensure that your manuscript meets PLOS ONE's style requirements, including those for file naming. The PLOS ONE style templates can be found at

https://journals.plos.org/plosone/s/file?id=wjVg/PLOSOne_formatting_sample_main_body.pdf and

https://journals.plos.org/plosone/s/file?id=ba62/PLOSOne_formatting_sample_title_authors_affiliations.pdf

2. To comply with PLOS One submissions requirements, in your Methods section, please provide additional information regarding the experiments involving animals and ensure you have included details on (1) methods of sacrifice, (2) methods of anesthesia and/or analgesia, and (3) efforts to alleviate suffering.

3. Please include your tables as part of your main manuscript and remove the individual files. Please note that supplementary tables (should remain/ be uploaded) as separate "supporting information" files.

4. We note that the grant information you provided in the ‘Funding Information’ and ‘Financial Disclosure’ sections do not match.

When you resubmit, please ensure that you provide the correct grant numbers for the awards you received for your study in the ‘Funding Information’ section.

5. Thank you for stating the following financial disclosure: [This research was funded by CNRS and in the frame of the AKC 2446 oak grant (“Development of Genetic Biomarkers to Improve Diagnosis and Treatment of Canine Histiocytic Sarcoma” 2018-2021), as well as by the AFBS donation in 2022 for WGS sequencing (Association Française des Bouviers Suisses). The sample collection performed through the French Cani-DNA BRC was funded by the CRB-Anim infrastructure ANR-11-INBS-0003 in the frame of the Investing for the Future Program (PIA1) and by the French Infrastructures en Biologie et Santé (IBiSA) specific BRC call (2022-2024)].

Please state what role the funders took in the study.  If the funders had no role, please state: ""The funders had no role in study design, data collection and analysis, decision to publish, or preparation of the manuscript.""

If this statement is not correct you must amend it as needed.

Please include this amended Role of Funder statement in your cover letter; we will change the online submission form on your behalf.

If the reviewer comments include a recommendation to cite specific previously published works, please review and evaluate these publications to determine whether they are relevant and should be cited. There is no requirement to cite these works unless the editor has indicated otherwise.

[Note: HTML markup is below. Please do not edit.]

Reviewers' comments:

Reviewer's Responses to Questions

**Comments to the Author**

1. Is the manuscript technically sound, and do the data support the conclusions?

The manuscript must describe a technically sound piece of scientific research with data that supports the conclusions. Experiments must have been conducted rigorously, with appropriate controls, replication, and sample sizes. The conclusions must be drawn appropriately based on the data presented.

Reviewer #1: Partly

Reviewer #2: Yes

**********

2. Has the statistical analysis been performed appropriately and rigorously?

Reviewer #1: I Don't Know

Reviewer #2: I Don't Know

**********

3. Have the authors made all data underlying the findings in their manuscript fully available?

The PLOS Data policy requires authors to make all data underlying the findings described in their manuscript fully available without restriction, with rare exception (please refer to the Data Availability Statement in the manuscript PDF file). The data should be provided as part of the manuscript or its supporting information, or deposited to a public repository. For example, in addition to summary statistics, the data points behind means, medians and variance measures should be available. If there are restrictions on publicly sharing data—e.g. participant privacy or use of data from a third party—those must be specified.

Reviewer #1: Yes

Reviewer #2: Yes

**********

4. Is the manuscript presented in an intelligible fashion and written in standard English?

PLOS ONE does not copyedit accepted manuscripts, so the language in submitted articles must be clear, correct, and unambiguous. Any typographical or grammatical errors should be corrected at revision, so please note any specific errors here.

Reviewer #1: Yes

Reviewer #2: Yes

**********

5. Review Comments to the Author

Please use the space provided to explain your answers to the questions above. You may also include additional comments for the author, including concerns about dual publication, research ethics, or publication ethics. (Please upload your review as an attachment if it exceeds 20,000 characters)

Reviewer #1: Overview:

This manuscript reports the molecular evaluation of histiocytic sarcoma (HS) in 38 dogs. The authors using digital PCR and Sanger sequencing to compare the distribution, abundance and divergence of specific sequence mutations in different lesions from dogs with the disseminated form of the disease (DHS). The study focused primarily on the PTPN11 gene, in which hotspot mutations have been reported previously in canine HS. The aim was to determine whether there is evidence for clonal heterogeneity of the disease within spatially separate abdominal and thoracic masses within an individual.

Three of the 38 dogs showed different PTPN11 mutations in abdominal versus thoracic tumors. Two additional dogs showed a mutation in one site that was not detected in the other. Results from the study were interpreted as showing that as many as 45% of DHS cases in dogs may represent polyclonal disease at the time of diagnosis. The authors suggest that their findings may challenge conventional understanding of DHS as a late-stage metastatic cancer developing from an initially localized disease. The identification of genetically distinct lesions within the same individual has particular significance for developing effective treatment strategies and prognostic tools for this cancer in dogs, and may warrant re-evaluation of DHS as a comparative model of human disease.

General comments:

The manuscript is very well written, and is broadly concise without lacking in detail; however, there are a few elements that are overly brief (e.g. see comments below on low-pass sequencing data).

The concept is interesting and has some level of novelty in the approach used, but largely incorporates well established techniques and principles.

The subject of this study is pertinent given that canine DHS is highly aggressive and notoriously challenging to treat effectively, and shows a particularly high incidence of genomic disruption compared to many other dog cancers. It is also remarkably prevalent in the Bernese mountain dog, which allows for targeted studies that reduce the ‘noise’ associated with heterogenous genetic backgrounds. It would seem highly logical that a polyclonal origin, coupled with a propensity for continued molecular evolution, would confound the ability to control the proliferation of distinct masses within an individual. In turn, the rarity of HS in people means that there is a critical need for appropriate model systems with which to elucidate the genomic component of the disease.

The focus on PTPN11 is logical given its role in upregulating the MAPK pathway in HS of both species, and the conservation of sequence mutations at equivalent hotspots in both canine and human tumors.

My primary concern relates to conclusions being based on the extrapolation of data from 3 of 38 dogs with divergent mutations of a gene in tumors from different anatomic sites. (Note that the ‘Methods’ section of the Abstract mentions that 38 dogs were studied, but the ‘Results’ section of the Abstract implies 28). From this, the authors estimate that 24-45% of disseminated HS cases in dogs may represent polyclonal disease at the time of diagnosis, which is a very broad range. While they provide the statistical process from which they drew this conclusion, it seems somewhat preliminary at this point. The Discussion does reflect the limitations of the study, but the Abstract reads more emphatically, and the title even more so.

It was unclear to me how non-neoplastic samples, mentioned in the Methods, were involved in this study. Was a germline sample from each dog screened with Sanger sequencing/digital PCR alongside the tumor samples from the same individuals?

I found it difficult to follow the process for evaluating the two PTPN11 mutation hotspots. For example, in Table 1:

- lung tissue for dog 10013 is shown as mutated in hotspot 1 using Sanger sequencing, but the same sample is shown as wild-type for that codon by digital PCR

- a lung sample from dog 2287 was wild-type for hotspot 1, and mutated in hotspot 2. In contrast, a liver sample from the same dog was mutated in hotspot 1 and wild-type in hotspot 2.

Dog 2287 was therefore classified as discordant with different mutations, but these results are only shown for the Sanger sequencing. Does this mean that neither of these samples were subsequently screened using digital PCR? Is it possible that both sites harbored both mutations, but with dissimilar allele frequencies such that only one was detectable with Sanger sequencing? Since this is the main focus of the study, I wondered why all samples were not analyzed with the more sensitive technique (i.e. digital PCR)?

In turn there is no mention of the relative level of sensitivity achieved by mutation screening. The sensitivity of Sanger sequencing is generally considered to be around 10-20%, but digital PCR is considerably more sensitive (depending on the quantity of input DNA and the criteria for classification of mutant vs. wild-type), and it should be possible to provide some indication of the lower limit that can be detected. Were suitable control samples run?

The data derived from whole genome sequencing of two cells lines from one dog is well presented and compelling. It would be very useful for this approach to be carried out in the future for other individuals in the study. There is, however, no direct mention of the low-pass sequencing data (and associated figures and tables) in the Results section - the section around line 311 should be moved to the Results.

There is very little discussion of how the findings of this study relate to the existing literature, and this should be expanded. There are several prior studies of other human and canine cancers that involve molecular comparison of multiple tumors from the same individual. Can the authors provide any insight into why an individual might develop multiple, clonally distinct lesions of the same cancer type within a short time period? Is there any precedent for this in other cancers/species? Does it point towards any particular etiology?

Specific comments:

Abstract/Introduction - there is no mention of the NGS/CNV component of the study, if space allows it would be useful to include a brief reference to this, as there is currently no lead-in (first mention is line 155, with no context given)

Line 92 - indicates collection of blood samples, but there is no mention of subsequent DNA extraction, and their number and use is not clear.

Line 94 refers to ‘mirror samples’, please clarify what is meant by this term.

Were any precautions taken to ensure that tumor samples were free of cross-contamination during tissue processing, and that there was minimal normal tissue in the sample? This is important when considering low abundance sequence mutations.

Line 95 - given the diagnostic challenges associated with canine HS, please provide a brief summary of diagnostic criteria. Was there any histological evidence that different tumors from the same individual were not of the same origin?

Line 98 - is it possible that the molecular divergence of these cell lines was an artifact of culture?

Line 115 - is it possible that any samples had low frequency mutations that did not show up with Sanger sequencing, but which were not screened for with digital PCR because Sanger sequencing detected a different mutation?

Line 155 - please clarify which tumor samples were analyzed with low-pass sequencing, and why these were chosen

Line 174 - this seems to be the first mention of any analysis of ‘normal’ samples, are these the blood samples mentioned in line 92?

Line 176 - please provide more info on how the copy number of the segments was estimated

Line 186 - how was the age of onset determined?

Can the authors comment on the wide range within the cohort (age 2 to 11 years old)? Is there any significance in that?

Lines 189-192 - this information should be moved to the Methods section.

Following the comment on line 115, were both digital PCR assays run on samples that appeared to be wild-type for a hotspot mutation seen in a different organ?

Lines 195-201 - this information would be more effective as a figure

Table 1 - what is the significance of the four sets of results shown in red text?

Table 2 - clarify that the ‘ID’ in the second column represents the code assigned to each dog

Supplementary figures - please annotate the DNA copy number information to delineate the chromosomes on the X axis, it is currently difficult to make comparisons between different samples

Reviewer #2: 1. The study presents the results of original research.

2. Results reported have not been published elsewhere.

3. Experiments, statistics, and other analyses are performed to a high technical standard and are described in sufficient detail.

4. Conclusions are presented in an appropriate fashion and are supported by the data.

I would soften your conclusions in the percentage of cases with independent clones. The study indicates 3/38 dogs and the statistical model/estimate is not what you have shown.

5. The article is presented in an intelligible fashion and is written in standard English.

6. The research meets all applicable standards for the ethics of experimentation and research integrity.

7. The article adheres to appropriate reporting guidelines and community standards for data availability.

Throughout the title and manuscript, rather than using the term polyclonal, independent clones or clonal diversity are more appropriate terms and used in other areas of the manuscript. This is still a neoplastic process and not reactive/inflammatory process which is typically the meaning of polyclonal. The number of clones is still very limited, not a Gaussian distribution of clones.

Line 20 Rather than using prognostic markers consider indicators.

Lone 88: Did you do anything with the blood from all the dogs? Looks like only the blood from the dog with cell lines was analyzed for germline mutations?

Line 92 Please indicate the number of dogs.

Line 94 I am not sure what you mean by ‘mirror’ samples.

Line 96 Pathologic diagnosis is strong when more than one pathologist reviews the case as this can be a challenging diagnosis and pathologists do not always agree. Was IHC used to confirm any cases?

Line 109 Section of sanger sequencing and ddPCR methods. From the references I don’t see any mention of how you have accounted for artifacts in formalin-fixed, paraffin-embedded FFPE tissue. I think it could also be useful to briefly explain the method along with the reference. One reference took me to another. The Hedan reference did not actually describe the method for sequences but instead referenced Hedan et al 2011.

Line 191 how many dogs had subsequent ddPCR

Line 258 clonal statuses in 25-48% is theoretical or a model rather than proving this percentage in this study. I think you can say that this study has shown that anatomically different tumors display independent PTPN11 clones in 3 of 38 cases. In the discussion you could go into the estimation but I don’t believe you have displayed this in this study.

**********

6. PLOS authors have the option to publish the peer review history of their article (what does this mean?). If published, this will include your full peer review and any attached files.

If you choose “no”, your identity will remain anonymous but your review may still be made public.

**Do you want your identity to be public for this peer review?**  For information about this choice, including consent withdrawal, please see our Privacy Policy.

Reviewer #1: No

Reviewer #2: No

**********

[NOTE: If reviewer comments were submitted as an attachment file, they will be attached to this email and accessible via the submission site. Please log into your account, locate the manuscript record, and check for the action link "View Attachments". If this link does not appear, there are no attachment files.]

To ensure your figures meet our technical requirements, please review our figure guidelines: https://journals.plos.org/plosone/s/figures

You may also use PLOS’s free figure tool, NAAS, to help you prepare publication quality figures: https://journals.plos.org/plosone/s/figures#loc-tools-for-figure-preparation.

NAAS will assess whether your figures meet our technical requirements by comparing each figure against our figure specifications.

# Author comment: Author response to Decision Letter 1

**DOI:** 10.1371/journal.pone.0345429.r002

28 May 2026

Dear Editor Douglas H. Thamm,

Thank you for reviewing this article and inviting us to submit a revised version. We would also like to thank the two reviewers for their relevant comments. We have taken in account their comments as well as your requirements. We have clarified all points in the article and addressed their comments in the point-by-point response below. The main change made to the method, in response to reviewer 1’s comments, and in order to clarify and simplify the strategy and the text, was to re-analyse all samples ( tumors and bloods samples) using ddPCR method- the most sensitive method available-, and to remove the Sanger sequencing step.

Reviewer #1 (Reviewer Comments to the Author)

The manuscript is very well written, and is broadly concise without lacking in detail; however, there are a few elements that are overly brief (e.g. see comments below on low-pass sequencing data).

The concept is interesting and has some level of novelty in the approach used, but largely incorporates well established techniques and principles. The subject of this study is pertinent given that canine DHS is highly aggressive and notoriously challenging to treat effectively, and shows a particularly high incidence of genomic disruption compared to many other dog cancers. It is also remarkably prevalent in the Bernese mountain dog, which allows for targeted studies that reduce the ‘noise’ associated with heterogenous genetic backgrounds. It would seem highly logical that a polyclonal origin, coupled with a propensity for continued molecular evolution, would confound the ability to control the proliferation of distinct masses within an individual. In turn, the rarity of HS in people means that there is a critical need for appropriate model systems with which to elucidate the genomic component of the disease.

The focus on PTPN11 is logical given its role in upregulating the MAPK pathway in HS of both species, and the conservation of sequence mutations at equivalent hotspots in both canine and human tumors.

My primary concern relates to conclusions being based on the extrapolation of data from 3 of 38 dogs with divergent mutations of a gene in tumors from different anatomic sites. (Note that the ‘Methods’ section of the Abstract mentions that 38 dogs were studied, but the ‘Results’ section of the Abstract implies 28). From this, the authors estimate that 24-45% of disseminated HS cases in dogs may represent polyclonal disease at the time of diagnosis, which is a very broad range. While they provide the statistical process from which they drew this conclusion, it seems somewhat preliminary at this point. The Discussion does reflect the limitations of the study, but the Abstract reads more emphatically, and the title even more so.

It is true that these results are preliminary and need to be confirmed. It is important to remain cautious about the frequency of this phenomenon, even though the estimates were calculated by applying the appropriate corrections to the usable samples (i.e., the 28 PTPN11 mutated samples). We have therefore amended the title as suggested, as well as the abstract. The Title becomes:” Investigation of disseminated canine histiocytic sarcoma reveals an independent clonal origin in a significant number of cases”

It was unclear to me how non-neoplastic samples, mentioned in the Methods, were involved in this study. Was a germline sample from each dog screened with Sanger sequencing/digital PCR alongside the tumor samples from the same individuals?

We agree that it should be clarified that non-neoplastic samples were used as germline reference samples to confirm the somatic origin of the PTPN11 mutations. We analysed them using Sanger sequencing, and all were tested negative. Following the reviewers’ suggestions regarding detection techniques (see below), we finally decided to test all these germline DNA samples by the ddPCR technique, they were all “PTN11 wild type” negative and we added sentences in the results (lines 254-257, in marked-up copy of the manuscript).

I found it difficult to follow the process for evaluating the two PTPN11 mutation hotspots. For example, in Table 1:

- lung tissue for dog 10013 is shown as mutated in hotspot 1 using Sanger sequencing, but the same sample is shown as wild-type for that codon by digital PCR

This was an error, we apologize and corrected the table1. The lung tissue from dog 10013 was not tested for hotspot 1 by ddPCR, as a mutation had already been detected by Sanger sequencing. To clarify the stratey and simplify the text, we analysed all samples ( tumors and bloods samples) only by the sensitive ddPCR method, and removed the Sanger sequencing step.

During this process we detected new positive samples corrected the Table 1 accordingly.

- a lung sample from dog 2287 was wild-type for hotspot 1, and mutated in hotspot 2. In contrast, a liver sample from the same dog was mutated in hotspot 1 and wild-type in hotspot 2.

Dog 2287 was therefore classified as discordant with different mutations, but these results are only shown for the Sanger sequencing. Does this mean that neither of these samples were subsequently screened using digital PCR? Is it possible that both sites harbored both mutations, but with dissimilar allele frequencies such that only one was detectable with Sanger sequencing? Since this is the main focus of the study, I wondered why all samples were not analyzed with the more sensitive technique (i.e. digital PCR)?

You are correct; we did not perform confirmatory ddPCR testing on samples that were tested positive by Sanger sequencing, since Sanger sequencing is less sensitive than ddPCR and a sample positive by Sanger sequencing has a minor allele frequency (MAF) > 0.1-0.2. As initially described in the Materials and Methods section, the identification of PTPN11 gene mutations in various tumor samples was first performed by Sanger sequencing. Subsequently, to improve the sensitivity of the PTPN11 mutations detection (E76K and G503V), we re-analysed Sanger negative samples by ddPCR. Despite the limited sensitivity of Sanger sequencing, this method has the advantage of identifying mutations without prior knowledge, such as E69 in dogs 6847 and 8843, contrary to ddPCR, which targets known mutations.

However, to answer your concern and simplify the reading, we performed the dddPCR on all samples on the 2 hotspots. This analysis revealed that some samples previously classified as wild-type by Sanger sequencing did, in fact, harbor low-frequency mutations. For example, we identified two additional discordant cases initially considered wild-type: samples 8357 and 17417, which carried the E76K mutation at low allele frequencies of 1.48% and 2.14%, respectively (Please See Table1). To simplify the understanding of the manuscript, we presented only the results obtained by ddPCR and removed the Sanger sequencing results. We then modified the methods (lines 145-150), the results (lines 254-257), and Table 1, the S2 Table, the abstract (line 36), introduction (line 96).

In turn there is no mention of the relative level of sensitivity achieved by mutation screening. The sensitivity of Sanger sequencing is generally considered to be around 10-20%, but digital PCR is considerably more sensitive (depending on the quantity of input DNA and the criteria for classification of mutant vs. wild-type), and it should be possible to provide some indication of the lower limit that can be detected. Were suitable control samples run?

You are correct: the sensitivity of Sanger sequencing ranges from 10% to 20%, whereas the ddPCR assay provides accurate estimates of fractional abundance down to 0.0005% for the BRAF V600E mutation (https://pmc.ncbi.nlm.nih.gov/articles/PMC6642310/). To define the sensitivity of our ddPCR assay, we determined the limit of detection by diluting the positive control DNA in a wild type DNA, the limit of detection for hotspot 1 and 2 were 0.096% and 0.2%. Regarding to the control samples, each ddPCR run was performed in the presence of the corresponding positive mutated samples and wild-type samples, as well as a blank control (water). We added sentences regarding the ddPCR method, its sensitivity as well as these controls in the “Materials and Methods” section (lines 152-163).

The data derived from whole genome sequencing of two cells lines from one dog is well presented and compelling. It would be very useful for this approach to be carried out in the future for other individuals in the study. There is, however, no direct mention of the low-pass sequencing data (and associated figures and tables) in the Results section - the section around line 311 should be moved to the Results.

We agree that NGS approaches such as whole genome sequencing or exome sequencing should be carried out in the future, indeed we mentioned this in the discussion (lines 453-455). As suggested, we moved the low pass sequencing data in the results at the beginning of chapter 3.3 (lines 332-341).

There is very little discussion of how the findings of this study relate to the existing literature, and this should be expanded. There are several prior studies of other human and canine cancers that involve molecular comparison of multiple tumors from the same individual. Can the authors provide any insight into why an individual might develop multiple, clonally distinct lesions of the same cancer type within a short time period? Is there any precedent for this in other cancers/species? Does it point towards any particular etiology?

As suggested, we expand the discussion section, by presenting information of other studies on cancer in humans and dogs and by proposing few hypotheses to explain this situation (lines 480, 487-513).

Specific comments:

Abstract/Introduction - there is no mention of the NGS/CNV component of the study, if space allows it would be useful to include a brief reference to this, as there is currently no lead-in (first mention is line 155, with no context given)

We agree and added sentence lines 44-46 in the abstract and lines 100-102 in the introduction.

Line 92 - indicates collection of blood samples, but there is no mention of subsequent DNA extraction, and their number and use is not clear.

We agree and added the information in the material and methods (line 142) and in the results (line 254).

Line 94 refers to ‘mirror samples’, please clarify what is meant by this term.

Were any precautions taken to ensure that tumor samples were free of cross-contamination during tissue processing, and that there was minimal normal tissue in the sample? This is important when considering low abundance sequence mutations.

Mirror samples refer to a macroscopic tumour divided into two parts: one preserved in RNAlater for genomic experiments, and the other in formalin for histological analysis. Since this step is performed by the treating veterinarian, we have no control over the process. Therefore, it is critical to use highly sensitive techniques, such as ddPCR, to detect low-abundance sequence mutations. This has been clarified in the Materials and Methods section (Lines 118-119).

Line 95 - given the diagnostic challenges associated with canine HS, please provide a brief summary of diagnostic criteria. Was there any histological evidence that different tumors from the same individual were not of the same origin?

All the cases included in this study originated from the routine diagnostic activities of French veterinary pathology laboratories that are partners and contributors to our research program. The initial diagnoses were made by specialized veterinary pathologists, either European or American board-certified. For inclusion in the present project, all samples were reviewed by an ECVP diplomate pathologist (JA), who has been working for 15 years on our research program dedicated to canine histiocytic sarcomas.

The histological criteria used to diagnose a sample as histiocytic sarcoma are:

• Diffuse, infiltrative proliferation of large neoplastic round to polygonal cells

• Marked cellular pleomorphism (anisocytosis, anisokaryosis)

• Abundant pale to eosinophilic cytoplasm

• Round to oval nuclei, often with prominent nucleoli

• High mitotic index (frequent mitoses, sometimes atypical)

• Tissue destruction with effacement of normal architecture

• Frequent necrosis and/or inflammation

• Possible hemophagocytosis (in some cases)

All cases, including the different tumor samples from the same individual, fulfilled these diagnostic criteria.

As a standard procedure in all our projects, and even when the initial and secondary histopathological evaluations were concordant and consistent with a diagnosis of histiocytic sarcoma, immunohistochemical detection of the histiocytic marker Iba1 (ionized calcium-binding adapter molecule 1) was performed as a confirmatory step. We precise this in the material and methods (lines 120-126). There as not histological evidence that different tumors from the same individual were not of the same origin.

Line 98 - is it possible that the molecular divergence of these cell lines was an artifact of culture?

We agree that prolonged culture could artificially create molecular differences through the accumulation of specific mutations in certain cell lines. However, common initiating mutations should still be present and detectable, as is the case with the PTPN11 gene mutations that have been identified. Given that there is only a single mutation between the two cell lines, we can clearly conclude that the two original tumors were independent.

Line 115 - is it possible that any samples had low frequency mutations that did not show up with Sanger sequencing, but which were not screened for with digital PCR because Sanger sequencing detected a different mutation?

We agree, and as mentioned earlier, all samples have now been analysed by ddPCR.

Line 155 - please clarify which tumor samples were analyzed with low-pass sequencing, and why these were chosen

We agree and add sentences in the result section lines 332-341.

Line 174 - this seems to be the first mention of any analysis of ‘normal’ samples, are these the blood samples mentioned in line 92?

As previously mentioned, we corrected the material and methods and the results to clarify the use of DNA from blood samples.

Line 176 - please provide more info on how the copy number of the segments was estimated

We added a sentence in the Material and Methods section, lines 238-240.

Line 186 - how was the age of onset determined?

Can the authors comment on the wide range within the cohort (age 2 to 11 years old)? Is there any significance in that?

The age of onset was calculated as the date of histological or cytological diagnosis minus the date of birth. To clarify this approach, we have added an explanatory sentence in the Materials and Methods section (lines 125-126).

Given that histiocytic sarcoma is a polyfactorial disease, the wide range in age of onset may reflect: the accumulation of predisposing mutations, as shown in Figure S6 of Hedan et al. (2021), where dogs with a higher number of risk alleles tend to have a shorter median lifespan due to an earlier onset; as well as the exposure to environmental risk factors, including chronic inflammation and the use of anti-inflammatory treatments, which have been shown to influence disease development (Ruple et al., 2016).

Lines 189-192 - this information should be moved to the Methods section.

As discussed previously, we remove all the information on Sanger sequencing, thus this information was deleted.

Following the comment on line 115, were both digital PCR assays run on samples that appeared to be wild-type for a hotspot mutation seen in a different organ?

Initially, we tested only the mutation observed in the different organs, but we have now tested all the samples at both PTPN11 hot spots.

Lines 195-201 - this information would be more effective as a figure

We agree and added a figure (Figure 1).

Table 1 - what is the significance of the four sets of results shown in red text?

The goal was to highlight discordant results and potentially polyclonal cases. Since this information appears in the last column, the red coloured text has been removed.

Table 2 - clarify that the ‘ID’

Attachment Submitted filename: Answer to reviewers for the article Mottier et al.docx

# Peer review: Decision Letter 1

**DOI:** 10.1371/journal.pone.0345429.r003

**Authors:** Douglas Thamm, Douglas Thamm

17 Jun 2026

PONE-D-26-10931R1

Investigation of disseminated canine histiocytic sarcoma reveals an independent clonal origin in a significant number of cases.

PLOS One

Dear Dr. Hédan,

Thank you for submitting your manuscript to PLOS ONE. After careful consideration, we feel that it has merit but does not fully meet PLOS ONE’s publication criteria as it currently stands. Therefore, we invite you to submit a revised version of the manuscript that addresses the additional points raised during the review process.

==============================

**Please address all reviewer comments.**

==============================

Please submit your revised manuscript by Aug 01 2026 11:59PM. If you will need more time than this to complete your revisions, please reply to this message or contact the journal office at plosone@plos.org. When you're ready to submit your revision, log on to https://www.editorialmanager.com/pone/ and select the 'Submissions Needing Revision' folder to locate your manuscript file.

Please include the following items when submitting your revised manuscript:

- A letter that responds to each point raised by the academic editor and reviewer(s). You should upload this letter as a separate file labeled 'Response to Reviewers'.
- A marked-up copy of your manuscript that highlights changes made to the original version. You should upload this as a separate file labeled 'Revised Manuscript with Track Changes'.
- An unmarked version of your revised paper without tracked changes. You should upload this as a separate file labeled 'Manuscript'.

If you would like to make changes to your financial disclosure, please include your updated statement in your cover letter. Guidelines for resubmitting your figure files are available below the reviewer comments at the end of this letter.

If applicable, we recommend that you deposit your laboratory protocols in protocols.io to enhance the reproducibility of your results. Protocols.io assigns your protocol its own identifier (DOI) so that it can be cited independently in the future. For instructions see: https://journals.plos.org/plosone/s/submission-guidelines#loc-laboratory-protocols. Additionally, PLOS ONE offers an option for publishing peer-reviewed Lab Protocol articles, which describe protocols hosted on protocols.io. Read more information on sharing protocols at https://plos.org/protocols?utm_medium=editorial-email&utm_source=authorletters&utm_campaign=protocols.

As the corresponding author, your ORCID iD is verified in the submission system and will appear in the published article. PLOS supports the use of ORCID, and we encourage all coauthors to register for an ORCID iD and use it as well. Please encourage your coauthors to verify their ORCID iD within the submission system before final acceptance, as unverified ORCID iDs will not appear in the published article. *Only*  the individual author can complete the verification step; PLOS staff *cannot*  verify ORCID iDs on behalf of authors.

We look forward to receiving your revised manuscript.

Kind regards,

Douglas H. Thamm, V.M.D.

Academic Editor

PLOS One

Journal Requirements:

1. If the reviewer comments include a recommendation to cite specific previously published works, please review and evaluate these publications to determine whether they are relevant and should be cited. There is no requirement to cite these works unless the editor has indicated otherwise.

2. Please review your reference list to ensure that it is complete and correct. If you have cited papers that have been retracted, please include the rationale for doing so in the manuscript text, or remove these references and replace them with relevant current references. Any changes to the reference list should be mentioned in the rebuttal letter that accompanies your revised manuscript. If you need to cite a retracted article, indicate the article’s retracted status in the References list and also include a citation and full reference for the retraction notice.

[Note: HTML markup is below. Please do not edit.]

Reviewers' comments:

Reviewer's Responses to Questions

**Comments to the Author**

1. If the authors have adequately addressed your comments raised in a previous round of review and you feel that this manuscript is now acceptable for publication, you may indicate that here to bypass the “Comments to the Author” section, enter your conflict of interest statement in the “Confidential to Editor” section, and submit your "Accept" recommendation.

Reviewer #1: (No Response)

**********

2. Is the manuscript technically sound, and do the data support the conclusions?

The manuscript must describe a technically sound piece of scientific research with data that supports the conclusions. Experiments must have been conducted rigorously, with appropriate controls, replication, and sample sizes. The conclusions must be drawn appropriately based on the data presented.

Reviewer #1: Partly

**********

3. Has the statistical analysis been performed appropriately and rigorously?

Reviewer #1: I Don't Know

**********

4. Have the authors made all data underlying the findings in their manuscript fully available?

The PLOS Data policy requires authors to make all data underlying the findings described in their manuscript fully available without restriction, with rare exception (please refer to the Data Availability Statement in the manuscript PDF file). The data should be provided as part of the manuscript or its supporting information, or deposited to a public repository. For example, in addition to summary statistics, the data points behind means, medians and variance measures should be available. If there are restrictions on publicly sharing data—e.g. participant privacy or use of data from a third party—those must be specified.

Reviewer #1: Yes

**********

5. Is the manuscript presented in an intelligible fashion and written in standard English?

PLOS ONE does not copyedit accepted manuscripts, so the language in submitted articles must be clear, correct, and unambiguous. Any typographical or grammatical errors should be corrected at revision, so please note any specific errors here.

Reviewer #1: Yes

**********

6. Review Comments to the Author

Please use the space provided to explain your answers to the questions above. You may also include additional comments for the author, including concerns about dual publication, research ethics, or publication ethics. (Please upload your review as an attachment if it exceeds 20,000 characters)

Reviewer #1: The questions I raised in my review of the original submission are largely addressed in this revision. The exceptions are as follows:

In my opinion the title still reads too emphatically based on the number of samples analyzed and the proportion of tumors that show the phenomenon in question. Ultimately the data are based on observations regarding a single mutation in just three cases, with the assumption that the mutational profile of the small region of tumor sampled is representative of the whole tumor.

The use of the word ‘significant’ in the title is my key concern. I would suggest something to the effect of “Identification of divergent PTPN11 mutations in canine histiocytic sarcomas reveals evidence of an independent clonal origin”. Similarly, I would suggest modifying the running title to “Evidence of clonal diversity origin in disseminated canine histiocytic sarcoma”.

Regarding control samples used for ddPCR analysis: If I understand correctly, the authors' comments suggest that the positive control for ddPCR was taken from among the panel of mutant-positive cases being tested for that mutation? I recommend including a statement indicating that this sample was first validated by Sanger sequencing as bearing the mutation, prior to being used as an internal positive control

Regarding the nature of blood samples mentioned: The revised text now reads “ddPCR assay included corresponding positive samples of tumors, wild-type samples, and a blank control (water)” - it is not immediately clear here that “wild type samples” refers to the patient-matched blood samples. Assuming that is the case, the term ‘wild type samples’ should be replaced by “matched blood samples from the same individuals” (or similar), since the ddPCR assay is being run to determine IF these samples are in fact wild-type.

The term ‘mirror sample’ is not one I have encountered before, and I notice the other viewer commented the same, so I recommend the text should include the definition given in the authors' response.

Regarding the possibility of molecular divergence during prolonged culture: I recommend including the authors' brief response to my query in the main text

The term 'age of onset' should be replaced by 'age at diagnosis'

**********

7. PLOS authors have the option to publish the peer review history of their article (what does this mean?). If published, this will include your full peer review and any attached files.

If you choose “no”, your identity will remain anonymous but your review may still be made public.

**Do you want your identity to be public for this peer review?**  For information about this choice, including consent withdrawal, please see our Privacy Policy.

Reviewer #1: No

**********

[NOTE: If reviewer comments were submitted as an attachment file, they will be attached to this email and accessible via the submission site. Please log into your account, locate the manuscript record, and check for the action link "View Attachments". If this link does not appear, there are no attachment files.]

To ensure your figures meet our technical requirements, please review our figure guidelines: https://journals.plos.org/plosone/s/figures

You may also use PLOS’s free figure tool, NAAS, to help you prepare publication quality figures: https://journals.plos.org/plosone/s/figures#loc-tools-for-figure-preparation.

NAAS will assess whether your figures meet our technical requirements by comparing each figure against our figure specifications.

# Author comment: Author response to Decision Letter 2

**DOI:** 10.1371/journal.pone.0345429.r004

24 Jun 2026

Dear Editor Douglas H. Thamm,

Thank you for reviewing this article and inviting us to submit a revised version. We would also like to thank the reviewers for their relevant comments. We have taken in account their comments and the remaining questions.

Reviewer #1 (Reviewer Comments to the Author)

The questions I raised in my review of the original submission are largely addressed in this revision. The exceptions are as follows:

In my opinion the title still reads too emphatically based on the number of samples analyzed and the proportion of tumors that show the phenomenon in question. Ultimately the data are based on observations regarding a single mutation in just three cases, with the assumption that the mutational profile of the small region of tumor sampled is representative of the whole tumor.

The use of the word ‘significant’ in the title is my key concern. I would suggest something to the effect of “Identification of divergent PTPN11 mutations in canine histiocytic sarcomas reveals evidence of an independent clonal origin”. Similarly, I would suggest modifying the running title to “Evidence of clonal diversity origin in disseminated canine histiocytic sarcoma”.

We have amended the titles as suggested.

Regarding control samples used for ddPCR analysis: If I understand correctly, the authors' comments suggest that the positive control for ddPCR was taken from among the panel of mutant-positive cases being tested for that mutation? I recommend including a statement indicating that this sample was first validated by Sanger sequencing as bearing the mutation, prior to being used as an internal positive control

We agree that it should be clarified, we corrected it lines 146-147.

Regarding the nature of blood samples mentioned: The revised text now reads “ddPCR assay included corresponding positive samples of tumors, wild-type samples, and a blank control (water)” - it is not immediately clear here that “wild type samples” refers to the patient-matched blood samples. Assuming that is the case, the term ‘wild type samples’ should be replaced by “matched blood samples from the same individuals” (or similar), since the ddPCR assay is being run to determine IF these samples are in fact wild-type.

We agree that it should be clarified, we corrected it line 147.

The term ‘mirror sample’ is not one I have encountered before, and I notice the other viewer commented the same, so I recommend the text should include the definition given in the authors' response.

You are correct the term was misleading; we have therefore removed it from the ‘Materials and Methods’ section in the latest version, whilst retaining the ‘definition’ in that same section: lines 107 to 109. “Each biopsy was divided into two parts, one was placed in RNALater solution for genomic experiments and the other was fixed in formalin for histopathological analyses, respectively. “

Regarding the possibility of molecular divergence during prolonged culture: I recommend including the authors' brief response to my query in the main text.

We added it in the discussion lines 355-359.

The term 'age of onset' should be replaced by 'age at diagnosis'

We corrected it Lines 114 and 229.

Attachment Submitted filename: Answer_to_reviewers_for_the_article_Mottier_et_al_auresp_2.docx

# Editor report: Decision Letter 2

**DOI:** 10.1371/journal.pone.0345429.r005

**Authors:** Douglas Thamm, Douglas Thamm, Douglas Thamm

25 Jun 2026

Identification of divergent PTPN11 mutations in canine histiocytic sarcomas reveals evidence of an independent clonal origin.

PONE-D-26-10931R2

Dear Dr. Hédan,

We’re pleased to inform you that your manuscript has been judged scientifically suitable for publication and will be formally accepted for publication once it meets all outstanding technical requirements.

Within one week, you’ll receive an e-mail detailing the required amendments. When these have been addressed, you’ll receive a formal acceptance letter and your manuscript will be scheduled for publication.

An invoice will be generated when your article is formally accepted. Please note, if your institution has a publishing partnership with PLOS and your article meets the relevant criteria, all or part of your publication costs will be covered. Please make sure your user information is up-to-date by logging into Editorial Manager at Editorial Manager® and clicking the ‘Update My Information' link at the top of the page. For questions related to billing, please contact billing support.

If your institution or institutions have a press office, please notify them about your upcoming paper to help maximize its impact. If they’ll be preparing press materials, please inform our press team as soon as possible -- no later than 48 hours after receiving the formal acceptance. Your manuscript will remain under strict press embargo until 2 pm Eastern Time on the date of publication. For more information, please contact onepress@plos.org.

Kind regards,

Douglas H. Thamm, V.M.D.

Academic Editor

PLOS One

Additional Editor Comments (optional):

Reviewers' comments:

# Editor report: Acceptance letter

**DOI:** 10.1371/journal.pone.0345429.r006

**Authors:** Douglas Thamm, Douglas Thamm, Douglas Thamm

PONE-D-26-10931R2

PLOS One

Dear Dr. Hédan,

I'm pleased to inform you that your manuscript has been deemed suitable for publication in PLOS One. Congratulations! Your manuscript is now being handed over to our production team.

At this stage, our production department will prepare your paper for publication. This includes ensuring the following:

* All references, tables, and figures are properly cited

* All relevant supporting information is included in the manuscript submission,

* There are no issues that prevent the paper from being properly typeset

You will receive further instructions from the production team, including instructions on how to review your proof when it is ready. Please keep in mind that we are working through a large volume of accepted articles, so please give us a few days to review your paper and let you know the next and final steps.

Lastly, if your institution or institutions have a press office, please let them know about your upcoming paper now to help maximize its impact. If they'll be preparing press materials, please inform our press team within the next 48 hours. Your manuscript will remain under strict press embargo until 2 pm Eastern Time on the date of publication. For more information, please contact onepress@plos.org.

You will receive an invoice from PLOS for your publication fee after your manuscript has reached the completed accept phase. If you receive an email requesting payment before acceptance or for any other service, this may be a phishing scheme. Learn how to identify phishing emails and protect your accounts at https://explore.plos.org/phishing.

If we can help with anything else, please email us at customercare@plos.org.

Thank you for submitting your work to PLOS ONE and supporting open access.

Kind regards,

PLOS ONE Editorial Office Staff

on behalf of

Dr. Douglas H. Thamm

Academic Editor

PLOS One
